# Automated Glycan Assembly of Oligogalactofuranosides Reveals the Influence of Protecting Groups on Oligosaccharide Stability

**DOI:** 10.1021/acs.joc.1c00505

**Published:** 2021-05-07

**Authors:** Narayana Murthy Sabbavarapu, Peter H. Seeberger

**Affiliations:** † Department of Biomolecular Systems, Max-Planck-Institute of Colloids and Interfaces, 14476 Potsdam, Germany; ‡ Freie Universität Berlin, Institute of Chemistry and Biochemistry, 14195 Berlin, Germany

## Abstract

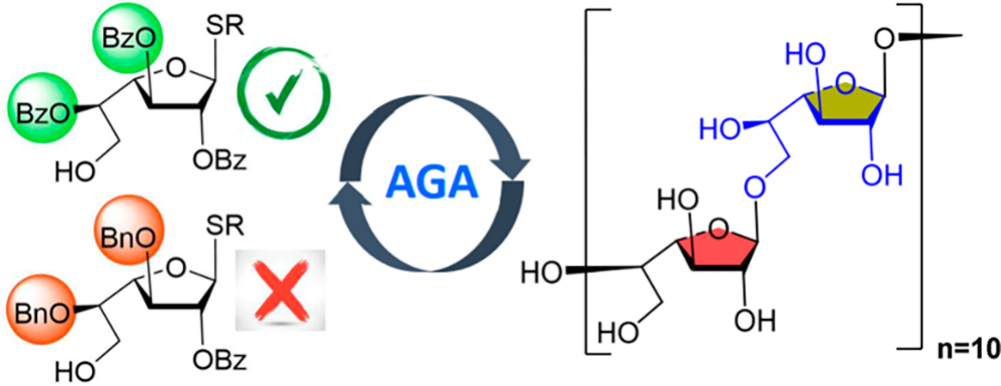

Galactofurans are an important structural constituent of arabinogalactan and lipopolysaccharides (LPS) ubiquitously present on the envelopes of all *Mycobacteria*. Key to the automated glycan assembly (AGA) of linear galactofuranosides as long as 20-mers was the identification of thioglycoside building blocks with a fine balance of stereoelectronic and steric effects to ensure the stability of oligogalactofuranoside during the synthesis. A benzoylated galactofuranose thioglycoside building block proved most efficient for oligosaccharide construction.

Tuberculosis caused by *Mycobacterium tuberculosis* (*M. tb.*), kills more people than any other infectious disease.^1^*M. tb.* bacteria are surrounded by an intricate network of mycoyl chains that form a dense outer hydrophobic framework that is critical for survival and pathogenicity of the organism.^2^ The TB cell wall consists of two major structural components, arabinogalactan (AG) and lipoarabinomannan (LAM) that are both composed of d-galactose and d-arabinose furanoses. Arabinogalactan consists of a linear galactan backbone of approximately 30 alternating β-(1 → 5)- and β-(1 → 6)-linked galactofuranose (Gal*f*) residues.^3^ Furanose-containing oligosaccharides are important for microorganisms, but rarely found in humans and other primates. Therefore, the enzymes that are necessary for the construction of galactofuranosyl motifs in microorganisms are attractive targets for the development of new antituberculosis drugs.^4^ The low abundance of bifunctional galactofuranosyltransferase (GlfT2) and the structural heterogeneity of oligogalactofuranosides limits the access to probes for cell-wall biosynthesis and to determine substrate specificities.^5^

Well-defined synthetic galactofuranosides that resemble the interior portion of AG are necessary to establish structure–activity relationships for these carbohydrates.^6^ Solution phase syntheses of galctofuranosyl oligomers ranging from 4 to 12 Gal*f* residues have been reported.^7^ A stepwise synthesis of a galactan tetramer revealed structural constraints in the trisaccharide nucleophile that resulted in drastically reduced reactivity. Therefore, a “nonreducing to reducing end” strategy relying on monosaccharide nucleophiles was employed, to prepare a tetrasaccharide galactan.^7a^ The synthesis of longer galctofuranosyl oligomers relied on an iterative glycosylation approach.^7b,8^ A range of galactofuranosides were synthesized to probe substrate specificities in biological systems.^9^ Most oligosaccharide sequences were prepared via stepwise syntheses that require many discrete operations and multiple purifications.

Automated glycan assembly was developed to accelerate oligosaccharide synthesis.^10^ Over the past two decades, it has been improved to access more complex glycans.^11^ However, oligofuranosides were not prepared by AGA beyond short arabinofuranosides.^12^ To explore the utility of AGA^11^ to prepare oligogalactofuranosides, we wanted to test the limits of preparing linear galactans found on the surface of *M. tb.* Here, we disclose the automated synthesis of linear oligogalactofuranoside 20-mer **1** using building blocks with judiciously selected orthogonal protecting groups (Figure 1).

**Figure 1:**
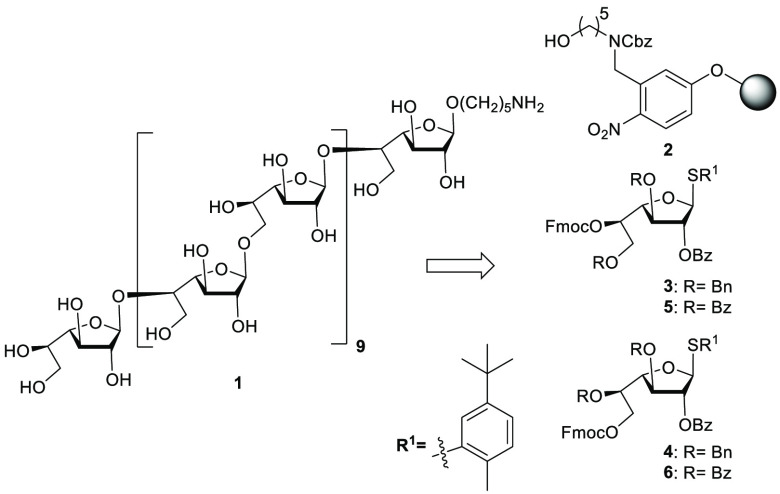
Retrosynthetic analysis of β-(1 → 5)- and β-(1 → 6)-linked linear galactan 20-mer **1**.

The power of automated synthesis relies on the section of differentially protected monosaccharide building blocks that result in high yielding and completely selective glycosylations. Galactofuranose thioglycoside building blocks were designed to carry a temporary 9-fluorenylmethoxycarbonyl (Fmoc) protecting group at C-5 (**3**) or C-6 (**4**) respectively. A C-2 benzoate provides anchimeric assistance to ensure stereoselectivity for trans-glycosidic linkages. Regioselective benzoylation of thioglycoside **7**(13) was followed by 3-*O*-benzylation and subsequent aqueous acetic acid mediated hydrolysis of acetonide protection afforded diol **8** (Scheme 1). Transacetalation of **8** with benzylaldehyde dimethyl acetal under acidic conditions preceded the regioselective opening of the benzylidene acetal using triethyl silane under acidic conditions before the C-5 hydroxyl was protected with Fmoc to furnish building block **3** in excellent yield. Building block **4** was prepared from **8** by selective protection as the corresponding TBDPS ether and 5-*O*-benzylation to access thiofuranoside **9**. Selective cleavage of the silyl ether using acetic acid buffered TBAF, followed by installation of the C-6 Fmoc provided building block **4** (Scheme 1).

**Scheme 1:**
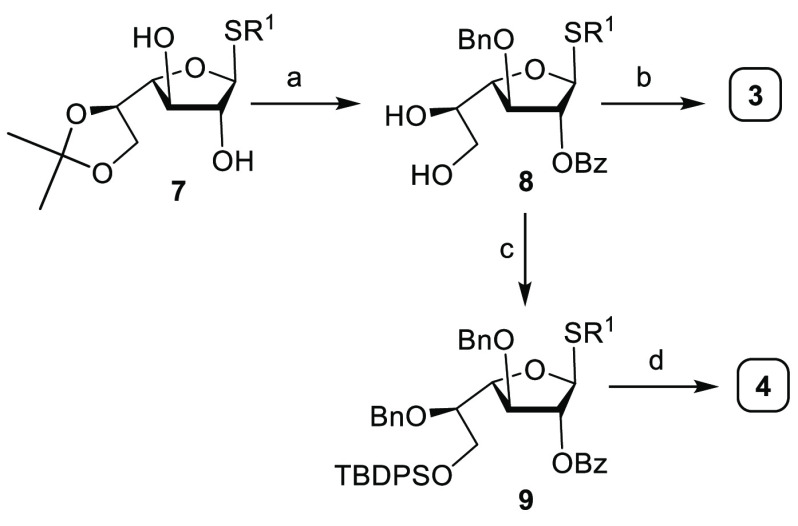
Synthesis of Building Blocks **3** and **4** Reagents and conditions: (a) (1) PhCOCl, Py., 46%; (2) Ag_2_O, BnBr, 78%; (3) AcOH/H_2_O, 80%; (b) (1) PhCH(OMe)_2_,CSA, 72%; (2) Et_3_SiH, TFA, TFAA, 82%; (3) FmocCl, Py., 87%; (c) (1) TBDPSCl, Im., 82%; (2) Ag_2_O, BnBr, 75%; (d) (1) TBAF, AcOH, 65%; (2) FmocCl, Py., 74%.

With thioglycoside building blocks **3** and **4** in hand, photocleavable aminopentanol linker immobilized on polystyrene resin **2** was placed in the reaction vessel of the automated synthesizer to prepare galactan heptamer **10** (Scheme 2). A four-step AGA process consisting of acidic wash, glycosylation, capping to mask unreacted nucleophiles, and removal of the temporary protecting group to expose the nucleophile for the next glycosylation was executed. UV irradiation using a continuous flow device released the protected oligosaccharide products from the polymer support that were analyzed using analytical HPLC and MALDI. In addition to desired galactan heptamer **10**, a host of deletion sequences were obtained. A careful analysis of the deletion sequences revealed that the temporary Fmoc protecting groups remained intact even after treatment with 20% piperidine in DMF. Changing the deprotection solution on the synthesizer to triethylamine (20% in DMF), or DBU (5% in DMF) and a higher reaction temperature (60 °C) failed to cleave Fmoc. The very hydrophobic Fmoc group may interact with hydrophobic regions of the sugar scaffold during oligosaccharide assembly to result in aggregation and poor reactivity.

**Scheme 2:**
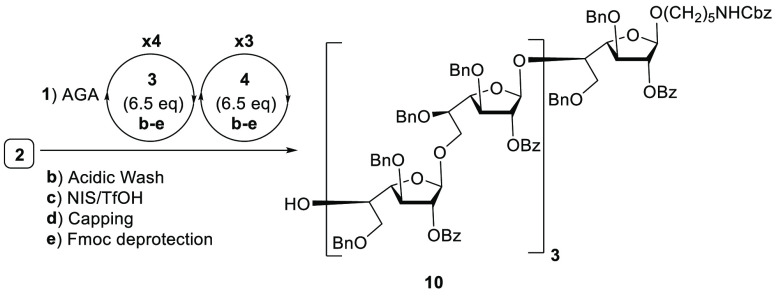
Synthesis of β-(1 → 5)- and β-(1 → 6)-Linked Linear Galactan Heptamer **10** Using Building Blocks **3** and **4**

To counteract aggregation and improve resin swelling, dichloromethane was used as solvent and the use of DBU (5% in CH_2_Cl_2_) resulted in complete Fmoc cleavage. However, AGA of galactan heptamer **10** using the improved deprotection step revealed unwanted deletion sequences with exposed hydroxyl groups. Apparently, the arming benzyl ethers at C-3, C-6 in building block **3** and C-3, C-5 positions in **4** have profound impact on the stability of the growing galactofuranoside due to intrinsic steric and stereoelectronic effects.^14^

On the basis of previous observations, we speculated that thiofuranosides **5** and **6** containing disarming benzoate esters may facilitate the assembly of linear oligogalactofuranoses. Building blocks **5** and **6** were prepared from thiofuranoside **7**(13) by benzoylation and isopropylidene cleavage to afford **11**. Regioselective benzoylation of **11** at low temperature and placement of Fmoc on the remaining secondary hydroxyl furnished **5**. Selective silylation of the 6-hydroxyl in **11** with TBDPSCl and benzoylation gave **12**. Desilylation of **12** by HF/pyridine followed by Fmoc protection yielded thioglycoside **6** (Scheme 3).

**Scheme 3:**
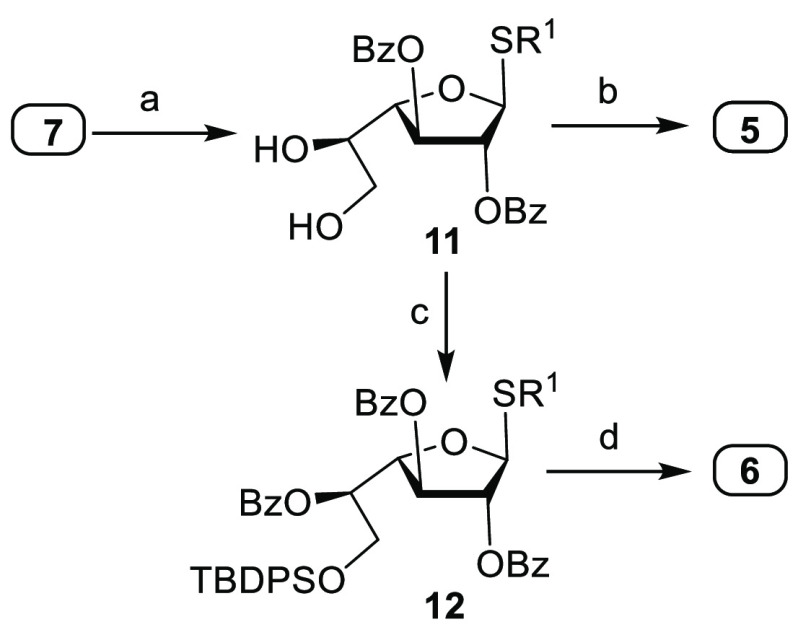
Synthesis of Building Blocks **5** and **6** Reagents and conditions: (a) (1) PhCOCl, Py., 86%; (2) AcOH/H_2_O, 80%; (b) (1) PhCOCl, Py., CH_2_Cl_2_, −60 °C, 82%; (2) FmocCl, Py., 89%; (c) (1) TBDPSCl, imidazole, 85%; (2) PhCOCl, Py., 78%; (d) (1) HF/Py., 72%; (2) FmocCl, Py., 80%.

AGA of galactan heptamer **13** using thiofuranosides **5** and **6** produced a single product according to the HPLC trace of the crude product (Scheme 4 and Figures S1 and S2). This encouraging result prompted us to prepare longer galactofuranose oligomers and to evaluate the influence of the protecting groups on the building blocks (Bn vs Bz) on the stability of growing oligogalactofuranoside. Therefore, using the AGA process developed for shorter sequences, linear galactan 20-mer **1** was assembled using building blocks **5** and **6**. HPLC and MALDI analysis of the crude mixture revealed that per-*O*-benzoylated furanoside glycosides **5** and **6** performed well. The desired product was purified by preparative HPLC and the structural integrity of protected galactofuranoside 20-mer **14** was confirmed by ^1^H, ^13^C NMR, as well as MALDI mass spectrometry (Scheme 5). Fully protected galactan **14** (17 mg) was treated with sodium methoxide to cleave all benzoate ester groups, followed by Pd(OH)_2_/C-catalyzed hydrogenolysis in the presence of hydrogen to cleave the Cbz group furnishing linear galactan 20-mer **1** (2 mg).

**Scheme 4:**
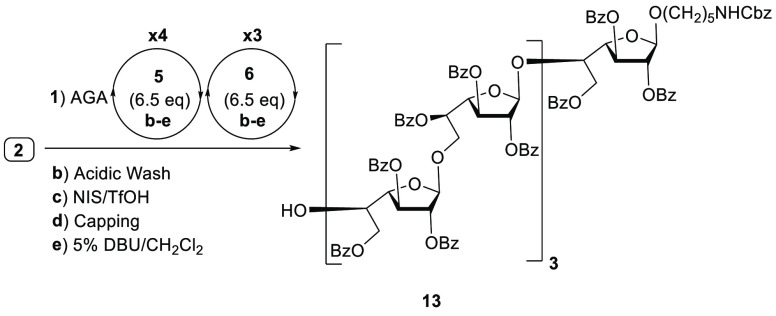
Synthesis of β-(1 → 5)- and β-(1 → 6)-Linked Linear Galactan Heptamer **13** Using Building Blocks **5** and **6**

**Scheme 5:**
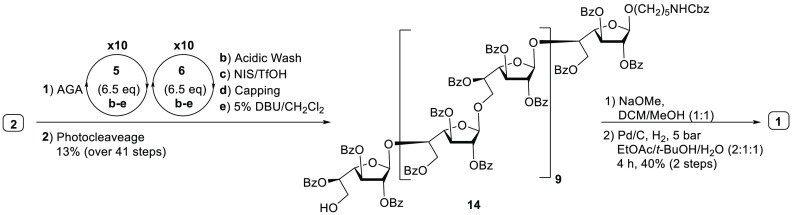
Synthesis of β-(1 → 5)- and β-(1 → 6)-Linked Linear Galactan 20-mer **1**

In conclusion, we disclose the first automated glycan assembly of oligogalactofuranosides. The identification of differentially protected benzoyl substituted galactofuranose thioglycoside building blocks was key to the successful automated synthesis of the glycans as long as 20-mers found on the cell surface of bacteria. The building blocks will be useful for the construction of many other oligofuranosides.

## Experimental Section

### General Information

All chemicals used were reagent grade and used as supplied unless otherwise noted. Automated syntheses were performed on a home-built synthesizer developed at the Max Planck Institute of Colloids and Interfaces.^15^ Merrifield resin LL (100–200 mesh, Novabiochem) was modified and used as solid support.^16^ Analytical thin-layer chromatography (TLC) was performed on Merck silica gel 60 F_254_ plates (0.25 mm). Compounds were visualized by UV irradiation or dipping the plate in a *p*-anisaldehyde (PAA) solution. Flash column chromatography was carried out by using forced flow of the indicated solvent on Fluka Kieselgel 60 M (0.04–0.063 mm). Analysis and purification by normal and reverse phase HPLC was performed using an Agilent 1200 series. Products were lyophilized using a Christ Alpha 2–4 LD plus freeze-dryer. ^1^H, ^13^C, and HSQC NMR spectra were recorded on a Varian 400-MR (400 MHz), Varian 600-MR (600 MHz), or Bruker Biospin AVANCE700 (700 MHz) spectrometer. Spectra were recorded in CDCl_3_ by using the solvent residual peak chemical shift as the internal standard (CDCl_3_: 7.26 ppm ^1^H, 77.16 ppm ^13^C) or in D_2_O using the solvent as the internal standard in ^1^H NMR (D_2_O: 4.79 ppm ^1^H) unless otherwise stated. High resolution mass spectra were obtained using a 6210 ESI-TOF mass spectrometer (Agilent) and a MALDI-TOF Autoflex (Bruker). MALDI and ESI mass spectra were run on IonSpec Ultima instruments.

### Automated Synthesis

Solvents used for dissolving building blocks and preparing the activator, TMSOTf, and capping solutions were taken from an anhydrous solvent system (jcmeyer-solvent systems). Other solvents used were HPLC grade. The building blocks were coevaporated three times with toluene and dried 2 h under a high vacuum before use. Activator, deprotection, acidic wash, capping, and building block solutions were freshly prepared and kept under argon during the automation run. All yields of products obtained by AGA were calculated based on resin loading. Resin loading was determined by performing one glycosylation (Module C) with ten equivalents of building block followed by DBU promoted Fmoc-cleavage and determination of dibenzofulvene production by measuring its UV absorbance.

### Preparation of Stock Solutions^17^

#### Building Block

Building block was dissolved in 1 mL dichloromethane (DCM).

#### Activator Solution

Recrystallized NIS (1.56 g) was dissolved in 60 mL of a 2:1 mixture of anhydrous CH_2_Cl_2_ and anhydrous dioxane. Then triflic acid (67 μL) was added. The solution was kept at 0 °C for the duration of the automation run.

#### Fmoc Deprotection Solution

A solution of 20% piperidine in dimethylformamide (DMF) (v/v) was prepared, or a solution of 5% DBU in dichloromethane (CH_2_Cl_2_) (v/v) was prepared.

#### TMSOTf Solution

Trimethylsilyl trifluoromethanesulfonate (TMSOTf) (0.9 mL) was added to DCM (80 mL).

#### Capping Solution

A solution of 10% acetic anhydride (Ac_2_O) and 2% methanesulfunic acid (MsOH) in anhydrous CH_2_Cl_2_ (v/v) was prepared.

### Modules for Automated Synthesis

#### Module A: Resin Preparation for Synthesis (20 min)

All automated syntheses were performed on 140 μmol scale (40 mg). Resin was placed in the reaction vessel and swollen in DCM for 20 min at room temperature prior to synthesis. During this time, all reagent lines required for the synthesis were washed and primed. Before the first glycosylation, the resin was washed with the DMF, tetrahydrofuran (THF), and CH_2_Cl_2_ (three times each with 2 mL for 25 s). This step is conducted as the first step for every synthesis.

#### Module B: Acidic Wash with TMSOTf Solution (20 min)

The resin was swollen in CH_2_Cl_2_ (2 mL) and the temperature of the reaction vessel was adjusted to −20 °C. Upon reaching the temperature, TMSOTf solution (1 mL) was added dropwise to the reaction vessel. After bubbling for argon 3 min, the acidic solution was drained and the resin was washed with 2 mL CH_2_Cl_2_ for 25 s.

#### Module C: Thioglycoside Glycosylation (20–60 min)

The building block solution (0.095–0.123 mmol (5–6.5 equiv) of BB in 1 mL of CH_2_Cl_2_ per glycosylation) was delivered to the reaction vessel. After the set temperature (−20 °C) was reached, the reaction was started by dropwise addition of the activator solution (1.0 mL, excess). The glycosylation was performed by increasing the temperature to 0 °C for 20–60 min (depending on oligosaccharide length). After completion of the reaction, the solution is drained and the resin was washed with CH_2_Cl_2_, CH_2_Cl_2_:dioxane (1:2, 3 mL for 20 s) and CH_2_Cl_2_ (twice, each with 2 mL for 25 s). The temperature of the reaction vessel is increased to 25 °C for the next module.

#### Module D: Capping (30 min)

The resin was washed with DMF (twice with 2 mL for 25 s) and the temperature of the reaction vessel was adjusted to 25 °C. Pyridine solution (2 mL, 10% in DMF) was delivered into the reaction vessel. After 1 min, the reaction solution was drained and the resin washed with CH_2_Cl_2_ (three times with 3 mL for 25 s). The capping solution (4 mL) was delivered into the reaction vessel. After 20 min, the reaction solution was drained and the resin washed with CH_2_Cl_2_ (three times with 3 mL for 25 s).

#### Module E: Fmoc Deprotection (14 min)

The resin was washed with DMF (three times with 2 mL for 25 s) and the temperature of the reaction vessel was adjusted to 25 °C. Fmoc deprotection solution (2 mL) was delivered into the reaction vessel. After 5 min, the reaction solution was drained and the resin washed with DMF (three times with 3 mL for 25 s) and CH_2_Cl_2_ (five times each with 2 mL for 25 s). The temperature of the reaction vessel is decreased to −20 °C for the next module.

### Postsynthesizer Manipulations

#### Cleavage from Solid Support

After automated synthesis, the oligosaccharides were cleaved from the solid support using a continuous-flow photo reactor as described previously.^11c,18^

#### Purification

Solvent was evaporated in vacuo and the crude products were dissolved in a 1:1 mixture of hexane and ethyl acetate and analyzed using analytical HPLC (DAD1F, 280 nm). Pure compounds were afforded by preparative HPLC (Agilent 1200 Series spectrometer).

#### Method A

(YMC-Diol-300 column, 150 × 4.6 mm) flow rate of 1.0 mL/min with Hex −20% EtOAc as eluents [isocratic 20% EtOAc (5 min), linear gradient to 60% EtOAc (5 min), linear gradient to 60% EtOAc (30 min), linear gradient to 100% EtOAc (5 min)].

#### Method B

(Synergi Hydro RP18 column, 250 × 10 mm) flow rate of 4.0 mL/min with water (0.1% formic acid) as eluents [isocratic (5 min), linear gradient to 10% ACN (30 min), linear gradient to 100% ACN (5 min)].

#### (2*S*,3*R*,4*R*,5*R*)-2-((5-(*tert*-Butyl)-2-methylphenyl)thio)-5-((*R*)-2,2-dimethyl-1,3-dioxolan-4-yl)tetrahydrofuran-3,4-diol (**7**)

Compound **7** (30.0 g) was prepared in 6 steps from d-Galactose (75.0 g, 416.29 mmol) following the literature procedures^7a,13^ as a light yellow sticky liquid.

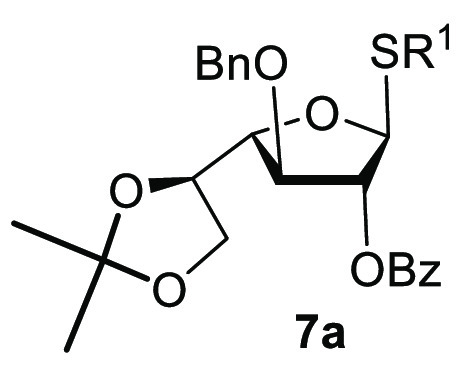



#### (2*S*,3*R*,4*R*,5*R*)-4-(Benzyloxy)-2-((5-(*tert*-butyl)-2-methylphenyl)thio)-5-((*R*)-2,2-dimethyl-1,3-dioxolan-4-yl)tetrahydrofuran-3-yl benzoate (**7a**)

To a stirred solution of **7**(13) (10.0 g, 26.14 mmol) in pyridine/CH_2_Cl_2_ (60 mL/600 mL) was added PhCOCl (3.34 mL, 28.74 mmol) dropwise at 0 °C, and the resulting mixture was gradually warmed to room temperature. The reaction mixture was stirred for 4 h at the same temperature, at the end of which time TLC indicated it was finished. The reaction was quenched with MeOH, diluted with CH_2_Cl_2_, and the mixture was washed with 1 M HCl, aq. NaHCO_3_, brine and dried over MgSO_4_. The combined organic layers were filtered, and concentrated. The residue was purified by silica gel column chromatography (ethyl acetate/*n*-hexanes: 20/80) to afford corresponding 2-*O*-benzoylated derivative in 46% yield (5.0 g) light-brown sticky liquid.

2-*O*-Benzoylated derivative (5.0 g, 10.27 mmol) from the above was dissolve in anhydrous CH_2_Cl_2_ containing 4 Å molecular sieves, was added silver oxide (4.76 g, 20.54 mmol) and BnBr (1.46 mL, 12.32 mmol) at 0 °C under nitrogen atmosphere. The reaction temperature was gradually warmed to room temperature, the solution was kept stirring for 48 h. After completion of the reaction as indicated by TLC, the resulting mixture was filtered through a pad of Celite, the filtrate was concentrated in a vacuum, the crude residue was purified (ethyl acetate/*n*-hexanes: 20/80) to yield titled compound **7a** in 78% yield (4.62 g) as a thick syrup. ^1^H NMR (400 MHz, CDCl_3_) δ 8.02–7.91 (m, 2H), 7.59–7.48 (m, 2H), 7.38 (t, *J* = 7.8 Hz, 2H), 7.33–7.29 (m, 2H), 7.28–7.12 (m, 4H), 7.06 (d, *J* = 8.2 Hz, 1H), 5.52 (d, *J* = 1.4 Hz, 2H), 4.81 (d, *J* = 11.9 Hz, 1H), 4.57 (d, *J* = 11.9 Hz, 1H), 4.37 (t, *J* = 5.6 Hz, 1H), 4.19 (td, *J* = 6.7, 5.4 Hz, 1H), 3.93 (dt, *J* = 5.6, 1.3 Hz, 1H), 3.86–3.66 (m, 2H), 2.37 (s, 3H), 1.32 (s, 3H), 1.26 (s, 3H), 1.22 (s, 9H); ^13^C{^1^H} NMR (100 MHz, CDCl_3_) δ 165.4, 149.6, 137.8, 137.2, 133.6, 132.3, 131.5, 130.0, 129.9, 129.4, 128.6, 128.6, 128.3, 128.1, 125.4, 109.8, 91.4, 83.7, 82.8, 82.3, 75.4, 72.5, 65.5, 34.5, 31.4, 29.8, 26.4, 25.5, 20.6; ESI HR-MS *m*/*z* [M + Na]^+^ calcd. for C_34_H_40_NaO_6_S: 599.2443, found 599.2464.

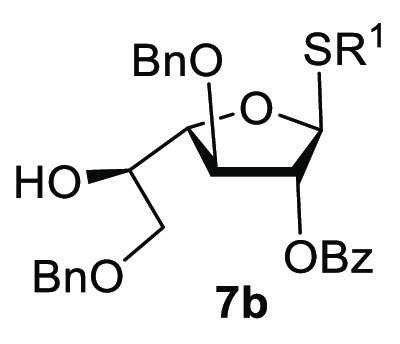



#### (2*S*,3*R*,4*R*,5*R*)-4-(Benzyloxy)-5-((*R*)-2-(benzyloxy)-1-hydroxyethyl)-2-((5-(*tert*-butyl)-2-methylphenyl)thio)tetrahydrofuran-3-yl benzoate (**7b**)

Compound **7a** (4.62 g, 8.01 mmol) was dissolved in 80% acetic acid in water and the mixture was stirred at 80 °C for 4 h. After completion of the reaction, the reaction mixture was concentrated and the residue was purified by column chromatography on silica gel (ethyl acetate/*n*-hexanes: 60/40) to give 5,6-diol **8** in 80% yield (3.44 g) as a colorless syrup. Then, diol (3.44 g, 6.41 mmol) was reacted with benzaldehyde dimethyl acetal (1.15 mL, 7.69 mmol) using CSA (0.22 g, 0.96 mmol) in the presence of CH_3_CN at room temperature. After completion of the reaction, Et_3_N was added, concentrated and purified by silica gel column chromatography to afford transacetalation product in 72% yield (2.88 g) colorless liquid.

The compound (2.88 g, 4.60 mmol) form the above step was dissolved in anhydrous CH_2_Cl_2_ containing 4 Å molecular sieves powder and stirred at room temperature for 15 min. After which, the reaction mixture was cooled to −78 °C and then Et_3_SiH (7.44 mL, 46.09 mmol) was added dropwise and stirred for 15 min. Then, TFA (3.52 mL, 46.09 mmol) was added and stirred for 15 min, followed by TFAA (0.12 mL, 0.92 mmol). The reaction mixture was stirred at −78 °C for 45 min, after which the reaction was removed from cooling bath and slowly brought to 0 °C. After being stirred for 1.5 h, the reaction was monitored by TLC, which indicated the completeness of the reaction. Then, the reaction mixture was filtered through a pad of Celite, the filtrate was washed with aq. NaHCO_3_, water, brine and concentrated in a vacuum, the crude residue was purified (ethyl acetate/*n*-hexanes: 20/80) to yield **7b** in 82% yield (2.36 g) as colorless thick syrup. ^1^H NMR (400 MHz, CDCl_3_) δ 7.98–7.92 (m, 2H), 7.54–7.47 (m, 2H), 7.37 (t, *J* = 7.8 Hz, 2H), 7.30–7.27 (m, 2H), 7.26–7.20 (m, 7H), 7.18 (d, *J* = 6.8 Hz, 1H), 7.13 (dd, *J* = 8.0, 2.1 Hz, 1H), 7.06 (d, *J* = 8.0 Hz, 1H), 5.54 (s, 1H), 5.51 (t, *J* = 1.7 Hz, 1H), 4.77 (d, *J* = 11.9 Hz, 1H), 4.55 (d, *J* = 11.9 Hz, 1H), 4.43 (d, *J* = 2.3 Hz, 2H), 4.39 (dd, *J* = 5.9, 3.0 Hz, 1H), 4.21 (ddd, *J* = 5.8, 1.9, 0.9 Hz, 1H), 3.92 (dt, *J* = 6.1, 2.6 Hz, 1H), 3.61–3.38 (m, 2H), 2.34 (s, 3H), 2.24 (d, *J* = 6.2 Hz, 1H), 1.20 (s, 9H).; ^13^C{^1^H} NMR (100 MHz, CDCl_3_) δ 165.5, 149.6, 137.8, 137.4, 137.2, 133.6, 132.8, 130.5, 130.0, 129.9, 129.3, 128.6, 128.6, 128.5, 128.1, 128.0, 127.9, 127.8, 125.1, 91.4, 83.3, 82.5, 82.4, 73.5, 72.6, 71.8, 69.7, 34.5, 31.4, 20.5; HR-MS *m*/*z* [M + Na]^+^ calcd. for C_38_H_42_NaO_6_S: 649.2600, found 649.2604.

#### (2*S*,3*R*,4*R*,5*R*)-5-((*R*)-1-((((9*H*-Fluoren-9-yl)methoxy)carbonyl)oxy)-2-(benzyloxy)ethyl)-4-(benzyloxy)-2-((5-(*tert*-butyl)-2-methylphenyl)thio)tetrahydrofuran-3-yl benzoate (**3**)

To a stirred solution of **7b** (2.36 g, 3.76 mmol) in anhydrous CH_2_Cl_2_ at 0 °C, FmocCl (2.43g, 9.41 mmol) and pyridine (1.51 mL, 18.82 mmol) were successively added and stirred at same temperature under ice bath for 4 h. After completion of the reaction as indicated by TLC, the reaction mixture was diluted with CH_2_Cl_2_ and washed with 1 M HCl, aq. NaHCO_3_, brine. The combined organic layers were dried over MgSO_4_, concentrated and purified by column chromatography using silica gel (ethyl acetate/*n*-hexanes: 20/80) to give **3** in 87% yield (2.78 g) as a white foam. ^1^H NMR (400 MHz, CDCl_3_) δ 8.03–7.94 (m, 2H), 7.67 (dd, *J* = 7.5, 1.1 Hz, 2H), 7.58–7.49 (m, 2H), 7.43 (ddd, *J* = 9.5, 7.0, 1.4 Hz, 2H), 7.37–7.25 (m, 6H), 7.21–7.09 (m, 11H), 7.04 (d, *J* = 8.0 Hz, 1H), 5.58 (s, 1H), 5.54 (t, *J* = 1.7 Hz, 1H), 5.21 (dt, *J* = 7.4, 4.5 Hz, 1H), 4.77 (d, *J* = 11.8 Hz, 1H), 4.58 (dd, *J* = 5.8, 4.0 Hz, 1H), 4.52 (d, *J* = 11.8 Hz, 1H), 4.48–4.36 (m, 2H), 4.27 (dd, *J* = 10.3, 7.7 Hz, 1H), 4.12 (dd, *J* = 10.3, 7.3 Hz, 1H), 4.08–4.01 (m, 2H), 3.65 (dd, *J* = 10.5, 7.3 Hz, 1H), 3.58 (dd, *J* = 10.6, 4.7 Hz, 1H), 2.34 (s, 3H), 1.20 (s, 9H); ^13^C{^1^H} NMR (100 MHz, CDCl_3_) δ 165.5, 155.1, 149.6, 143.5, 143.3, 141.3, 141.3, 137.7, 137.2, 137.1, 133.6, 132.7, 130.4, 130.0, 130.0, 129.2, 128.6, 128.5, 128.4, 128.2, 128.0, 127.9, 127.7, 127.6, 127.3, 127.2, 125.3, 125.3, 125.1, 120.1, 91.2, 83.1, 82.2, 81.1, 75.0, 73.3, 72.7, 70.2, 68.9, 46.7, 34.5, 31.4, 20.5; ESI HR-MS *m*/*z* [M + Na]^+^ calcd. for C_53_H_52_NaO_8_S: 871.3281, found 871.3297.

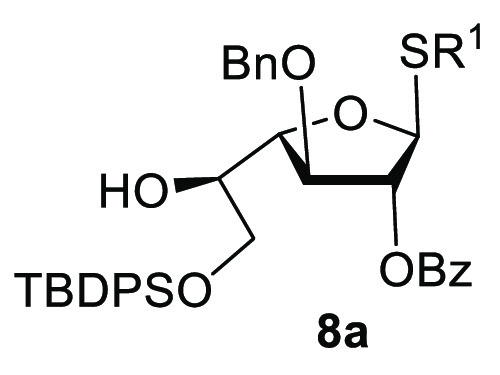



#### (2*S*,3*R*,4*R*,5*R*)-4-(Benzyloxy)-2-((5-(*tert*-butyl)-2-methylphenyl)thio)-5-((*R*)-2-((*tert*-butyldiphenylsilyl)oxy)-1-hydroxyethyl)tetrahydrofuran-3-yl benzoate (**8a**)

Compound **8** (6.6 g, 12.29 mmol) was dissolved in anhydrous CH_2_Cl_2_ and cooled to 0 °C. *tert*-Butyldiphenylsilyl chloride (5.5 mL, 13.52 mmol) was added dropwise, followed by the addition imidazole (2.09 g, 30.74 mmol). The reaction mixture was allowed to attain the room temperature under stirring, and the reaction was monitored by TLC, which indicated the completion after 3.5 h. The reaction was diluted with CH_2_Cl_2_ and water, and the two layers were separated. The aqueous layer was thoroughly washed with CH_2_Cl_2_and the combined organic layers were washed with brine solution and dried over anhydrous MgSO_4_. The solvent was evaporated to dryness and the residue was subjected to column chromatography (ethyl acetate/*n*-hexanes: 30/70) to yield corresponding silyl ether **8a** in 82% yield (7.81 g) as a thick syrup. ^1^H NMR (400 MHz, CDCl_3_) δ 8.05–7.98 (m, 2H), 7.68–7.53 (m, 6H), 7.50–7.27 (m, 13H), 7.18 (dd, *J* = 8.0, 2.1 Hz, 1H), 7.10 (d, *J* = 8.0 Hz, 1H), 5.63–5.52 (m, 2H), 4.83 (d, *J* = 11.9 Hz, 1H), 4.60 (d, *J* = 11.9 Hz, 1H), 4.50 (dd, *J* = 5.9, 2.8 Hz, 1H), 4.23 (dt, *J* = 5.7, 1.4 Hz, 1H), 3.85 (brs, 1H), 3.79–3.65 (m, 2H), 2.38 (s, 3H), 1.22 (s, 9H), 1.06 (s, 9H); ^13^C{^1^H} NMR (100 MHz, CDCl3) δ 165.5, 149.6, 137.5, 137.1, 135.6, 135.6, 133.6, 133.2, 133.21, 132.9, 130.3, 130.0, 129.9, 129.4, 128.6, 128.5, 128.0, 127.9, 125.0, 91.6, 83.6, 82.5, 82.0, 72.6, 71.2, 65.3, 34.5, 31.4, 29.8, 26.9, 20.5,19.3; ESI HR-MS *m*/*z* [M + Na]^+^ calcd. for C_47_H_54_NaO_6_SSi: 797.3308, found 797.3293.

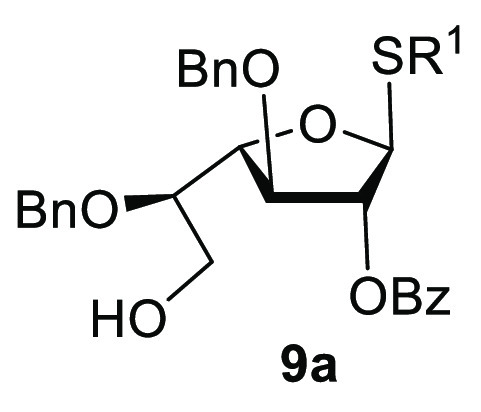



#### (2*S*,3*R*,4*R*,5*R*)-4-(Benzyloxy)-5-((*R*)-1-(benzyloxy)-2-hydroxyethyl)-2-((5-(*tert*-butyl)-2-methylphenyl)thio)tetrahydrofuran-3-yl benzoate (**9a**)

To a stirred solution of silyl ether **8a** (7.81 g, 10.08 mmol) in anhydrous CH_2_Cl_2_ containing 4 Å molecular sieves, was added silver oxide (4.66 g, 20.15 mmol) and BnBr (3.59 mL, 30.22 mmol) at 0 °C under nitrogen atmosphere. The reaction temperature was gradually warmed to room temperature, the solution was kept stirring for 48 h. After completion of the reaction as indicated by TLC, the resulting mixture was filtered through a pad of Celite, the filtrate was concentrated in a vacuum, the crude residue was purified (ethyl acetate/*n*-hexanes: 20/80) to yield corresponding benzoylated derivative in 75% yield (6.53 g) as a light-yellow syrup. Then, the fully protected compound (6.53 g, 7.55 mmol) was treated with 1 M TBAF (17.48 mL, 60.37 mmol) buffered with AcOH (1.72 mL, 30.18 mmol) in anhydrous THF at 0 °C for 4.5 h. After completion of the reaction as indicated by TLC, the reaction mixture was diluted with ethyl acetate and washed with water, brine and dried over anhydrous MgSO_4_. The combined organic layers were evaporated to dryness and subjected to column chromatography using silica gel (ethyl acetate/*n*-hexanes: 30/70) to yield **9a** in 65% yield (3.07 g) as a white foam. ^1^H NMR (400 MHz, CDCl_3_) δ 8.13–7.94 (m, 2H), 7.65–7.57 (m, 2H), 7.51–7.41 (m, 2H), 7.39–7.27 (m, 5H), 7.22 (ddt, *J* = 8.3, 4.6, 2.2 Hz, 6H), 7.14 (d, *J* = 8.0 Hz, 1H), 5.65 (q, *J* = 1.0 Hz, 1H), 5.56 (t, *J* = 1.6 Hz, 1H), 4.81 (d, *J* = 11.8 Hz, 1H), 4.65–4.56 (m, 2H), 4.49 (dd, *J* = 20.0, 11.7 Hz, 2H), 4.24–4.14 (m, 1H), 3.87–3.62 (m, 3H), 2.43 (s, 3H), 1.95 (dd, *J* = 7.9, 4.5 Hz, 1H), 1.28 (s, 9H); ^13^C{^1^H} NMR (100 MHz, CDCl_3_) δ 165.5, 149.7, 138.0, 137.2, 137.2, 133.6, 132.6, 130.2, 130.1, 129.9, 129.3, 128.6, 128.6, 128.5, 128.4, 128.2, 128.1, 127.9, 125.2, 91.1, 83.1, 83.1, 82.5, 73.1, 72.5, 62.5, 34.5, 31.4, 20.5; ESI HR-MS *m*/*z* [M + Na]^+^ calcd. for C_38_H_42_NaO_6_S: 649.2600, found 649.2613.

#### (2*S*,3*R*,4*R*,5*R*)-5-((*R*)-2-((((9*H*-Fluoren-9-yl)methoxy)carbonyl)oxy)-1-(benzyloxy)ethyl)-4-(benzyloxy)-2-((5-(*tert*-butyl)-2-methylphenyl)thio)tetrahydrofuran-3-yl benzoate (**4**)

To a stirred solution of **9a** (3.0 g, 4.78 mmol) in anhydrous CH_2_Cl_2_ at 0 °C, FmocCl (3.09 g, 11.96 mmol) and pyridine (1.92 mL, 23.93 mmol) were successively added and stirred at same temperature under ice bath for 4 h. After completion of the reaction as indicated by TLC, the reaction mixture was diluted with CH_2_Cl_2_ and washed with 1 M HCl, aq. NaHCO_3_, brine. The combined organic layers were dried over MgSO_4_, concentrated and purified by column chromatography using silica gel (ethyl acetate/*n*-hexanes: 20/80) to give **4** in 74% yield (3.0 g) as a white foam. ^1^H NMR (400 MHz, CDCl_3_) δ 7.97–7.90 (m, 2H), 7.73–7.68 (m, 2H), 7.57–7.47 (m, 4H), 7.34 (td, *J* = 7.7, 3.2 Hz, 4H), 7.31–7.20 (m, 7H), 7.14–7.08 (m, 6H), 7.04 (d, *J* = 8.0 Hz, 1H), 5.58 (s, 1H), 5.49 (t, *J* = 1.6 Hz, 1H), 4.72 (d, *J* = 11.9 Hz, 1H), 4.59 (d, *J* = 11.4 Hz, 1H), 4.51 (dd, *J* = 6.1, 3.2 Hz, 1H), 4.44–4.23 (m, 6H), 4.15 (t, *J* = 7.4 Hz, 1H), 4.07 (dt, *J* = 6.1, 1.3 Hz, 1H), 3.80 (ddd, *J* = 6.9, 5.1, 3.2 Hz, 1H), 2.33 (s, 3H), 1.21 (s, 9H); ^13^C{^1^H} NMR (100 MHz, CDCl_3_) δ 165.5, 155.0, 149.7, 143.4, 141.4, 137.69 137.4, 137.0, 133.5, 132.8, 130.1, 130.0, 129.3, 128.6, 128.6, 128.4, 128.4, 128.3, 128.1, 128.0, 127.9, 127.3, 127.3, 125.3, 125.0, 120.2, 91.3, 82.8, 82.7, 82.0, 77.3, 75.0, 73.7, 72.5, 70.0, 67.6, 46.7, 34.5, 31.4, 29.8, 20.5; ESI HR-MS *m*/*z* [M + Na]^+^ calcd. for C_53_H_52_NaO_8_S: 871.3281, found 871.3293.

#### (2*S*,3*R*,4*R*,5*R*)-2-((5-(*tert*-Butyl)-2-methylphenyl)thio)-5-((*R*)-1,2-dihydroxyethyl)tetrahydrofuran-3,4-diyl dibenzoate (**11**)^13^

To a solution of **7**(13) (5.0 g, 13.07 mmol) in pyridine was added PhCOCl (3.34 mL, 28.75 mmol) dropwise at 0 °C, and the resulting mixture was gradually warmed to room temperature. The reaction mixture was stirred for 4 h at the same temperature, at the end of which time TLC indicated it was finished. The reaction was quenched with MeOH, diluted with CH_2_Cl_2_, and the mixture was washed with 1 M HCl, aq. NaHCO_3_, brine and dried over MgSO_4_. The combined organic layers were filtered, and concentrated. The residue was purified by silica gel column chromatography (ethyl acetate/*n*-hexanes: 20/80) to afford corresponding 2,3-*O*-benzoylated derivative in 86% yield (6.64 g) as a glassy liquid.

To a stirred solution of 2,3-*O*-benzoylated derivative (6.64 g, 11.24 mmol) in 80% aqueous acetic acid was stirred at 80 °C for 5 h. After completion of the reaction, the reaction mixture was concentrated and the residue was purified by column chromatography on silica gel (ethyl acetate/*n*-hexanes: 60/40) to give **11** in 80% yield (4.95 g) as a colorless syrup. ^1^H NMR (400 MHz, CDCl_3_) δ 8.17–8.11 (m, 2H), 8.09–8.03 (m, 2H), 7.69–7.55 (m, 3H), 7.53–7.42 (m, 4H), 7.30–7.23 (m, 1H), 7.18 (d, *J* = 8.0 Hz, 1H), 5.74 (t, *J* = 1.6 Hz, 1H), 5.70 (dd, *J* = 4.3, 1.5 Hz, 2H), 4.59 (ddd, *J* = 5.2, 3.0, 0.9 Hz, 1H), 4.18 (q, *J* = 4.2 Hz, 1H), 3.96–3.70 (m, 2H), 2.73 (d, *J* = 7.9 Hz, 1H), 2.46 (s, 3H), 1.30 (s, 9H); ^13^C{^1^H} NMR (100 MHz, CDCl_3_) δ 166.1, 165.4, 149.9, 137.6, 133.9, 133.8, 131.9, 131.0, 130.3, 130.2, 130.0, 129.0, 129.0, 128.7, 128.7, 125.7, 91.4, 84.3, 82.1, 78.2, 77.3, 70.5, 64.4, 34.5, 31.4, 20.6; ESI HR-MS *m*/*z* [M + Na]^+^ calcd. for C_31_H_34_NaO_7_S: 573.1923, found 573.1910.

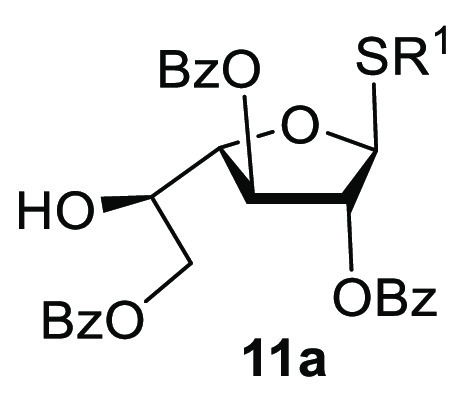



#### (2*R*,3*R*,4*R*,5*S*)-2-((*R*)-2-(Benzoyloxy)-1-hydroxyethyl)-5-((5-(*tert*-butyl)-2-methylphenyl)thio)tetrahydrofuran-3,4-diyl dibenzoate (**11a**)^13^

Compound **11** (2.77 g, 5.03 mmol) was dissolved in anhydrous CH_2_Cl_2_ and cooled to −60 °C. At this temperature, pyridine (2.02 mL, 25.15 mmol) was added and stirred for 5 min. Then, PhCOCl (0.65 mL, 5.63 mmol) was added dropwise and stirred for 30 min at −60 °C. The reaction progress was monitored by TLC. After 0.5 h, the reaction was completed and MeOH was added to quench the reaction. The reaction mixture was diluted CH_2_Cl_2_ and washed with 1 M HCl, aq. NaHCO_3_, brine and dried over MgSO_4_. The combined organic layers were filtered, and concentrated. The residue was purified by silica gel column chromatography (ethyl acetate/*n*-hexanes: 20/80) to afford **11a** in 82% yield (2.70 g) as a light-brown sticky liquid. ^1^H NMR (400 MHz, CDCl_3_) δ 8.17–8.11 (m, 2H), 8.08–7.98 (m, 4H), 7.68–7.35 (m, 10H), 7.24 (dd, *J* = 8.0, 2.1 Hz, 1H), 7.15 (d, *J* = 8.0 Hz, 1H), 5.78 (t, *J* = 1.6 Hz, 1H), 5.77–5.73 (m, 2H), 4.67 (dd, *J* = 4.6, 2.3 Hz, 1H), 4.61–4.49 (m, 2H), 4.45 (dd, *J* = 10.2, 3.8 Hz, 1H), 2.66 (d, *J* = 7.8 Hz, 1H), 2.45 (s, 3H), 1.29 (s, 9H); ^13^C{^1^H} NMR (100 MHz, CDCl_3_) δ 166.5, 166.1, 165.4, 149.9, 137.5, 133.8, 133.7, 133.2, 131.9, 131.0, 130.2, 130.2, 130.0, 129.8, 129.0, 129.0, 128.7, 128.7, 128.5, 125.6, 91.7, 83.2, 82.1, 78.3, 77.3, 69.1, 66.0, 34.5, 31.4, 20.6;ESI HR-MS *m*/*z* [M + Na]^+^ calcd. for C_38_H_38_NaO_8_S: 677.2185, found 677.2191.

#### (2*R*,3*R*,4*R*,5*S*)-2-((*R*)-1-((((9*H*-Fluoren-9-yl)methoxy)carbonyl)oxy)-2-(benzoyloxy)ethyl)-5-((5-(*tert*-butyl)-2-methylphenyl)thio)tetrahydrofuran-3,4-diyl dibenzoate (**5**)

To a stirred solution of **11a**(13) (2.7 g, 4.12 mmol) in anhydrous CH_2_Cl_2_ at 0 °C, FmocCl (2.66 g, 10.31 mmol) and pyridine (1.66 mL, 20.614 mmol) were successively added and stirred at same temperature under ice bath for 4 h. After completion of the reaction as indicated by TLC, the reaction mixture was diluted with CH_2_Cl_2_ and washed with 1 M HCl, aq. NaHCO_3_, brine. The combined organic layers were dried over MgSO_4_, concentrated and purified by column chromatography using silica gel (ethyl acetate/*n*-hexanes: 20/80) to give **5** in 89% yield (3.21 g) as white foam: ^1^H NMR (400 MHz, CDCl_3_) δ 8.13 (ddd, *J* = 8.4, 2.4, 1.3 Hz, 4H), 7.98–7.92 (m, 2H), 7.75 (d, *J* = 7.6 Hz, 2H), 7.68 (d, *J* = 2.1 Hz, 1H), 7.65–7.58 (m, 2H), 7.54–7.43 (m, 5H), 7.42–7.32 (m, 4H), 7.31–7.19 (m, 5H), 7.16 (d, *J* = 8.0 Hz, 1H), 5.80 (d, *J* = 1.4 Hz, 2H), 5.77 (dt, *J* = 7.8, 3.9 Hz, 1H), 5.67 (dt, *J* = 4.8, 1.3 Hz, 1H), 4.87 (t, *J* = 4.3 Hz, 1H), 4.75 (dd, *J* = 11.9, 4.0 Hz, 1H), 4.65 (dd, *J* = 12.0, 7.7 Hz, 1H), 4.38 (dd, *J* = 10.4, 8.1 Hz, 1H), 4.25 (dd, *J* = 10.4, 7.3 Hz, 1H), 4.18–4.09 (m, 1H), 2.46 (s, 3H), 1.30 (s, 9H); ^13^C{^1^H} NMR (100 MHz, CDCl_3_) δ 166.0, 165.7, 165.4, 155.0, 149.9, 143.5, 143.1, 141.3, 141.2, 137.4, 133.8, 133.7, 133.2, 132.0, 130.8, 130.2, 130.1, 129.8, 129.5, 129.0, 128.9, 128.7, 128.6, 128.4, 128.0, 127.9, 127.3, 127.3, 125.5, 125.4, 125.3, 120.1, 120.1, 91.5, 82.0, 81.6, 77.8, 77.3, 74.2, 70.6, 63.4, 46.6, 34.5, 31.4, 20.6; ESI HR-MS *m*/*z* [M + Na]^+^ calcd. for C_53_H_48_NaO_10_S: 899.2866, found 899.2877.

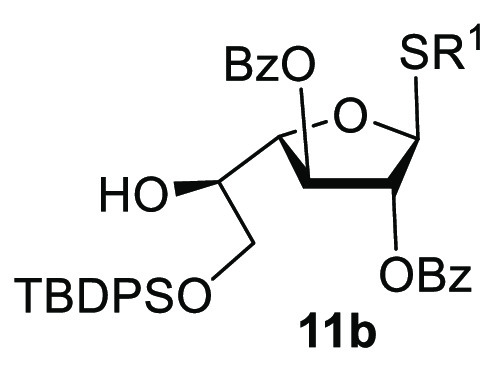



#### (2*S*,3*R*,4*R*,5*R*)-2-((5-(*tert*-Butyl)-2-methylphenyl)thio)-5-((*R*)-2-((*tert*-butyldiphenylsilyl)oxy)-1-hydroxyethyl)tetrahydrofuran-3,4-diyl dibenzoate (**11b**)

Compound **11** (2.5 g, 4.54 mmol) was dissolved in anhydrous CH_2_Cl_2_ and cooled to 0 °C. *tert*-Butyldiphenylsilyl chloride (1.29 mL, 4.99 mmol) was added dropwise, followed by the addition imidazole (0.77 g, 11.35 mmol). The reaction mixture was allowed to attain the room temperature under stirring, and the reaction was monitored by TLC, which indicated the completion after 3.5 h. The reaction was diluted with CH_2_Cl_2_ and water, and the two layers were separated. The aqueous layer was thoroughly washed with CH_2_Cl_2_and the combined organic layers were washed with brine solution and dried over anhydrous MgSO_4_. The solvent was evaporated to dryness and the residue was subjected to column chromatography (ethyl acetate/*n*-hexanes: 30/70) to yield corresponding silyl ether **11b** in 85% yield (3.04 g) as a colorless liquid: ^1^H NMR (400 MHz, CDCl_3_) δ 8.17–8.11 (m, 2H), 8.08–8.02 (m, 2H), 7.68–7.54 (m, 7H), 7.50 (t, *J* = 7.8 Hz, 2H), 7.46–7.36 (m, 4H), 7.32 (td, *J* = 7.0, 1.3 Hz, 4H), 7.21 (dd, *J* = 8.0, 2.1 Hz, 1H), 7.13 (d, *J* = 8.0 Hz, 1H), 5.75 (ddd, *J* = 5.1, 2.0, 0.9 Hz, 1H), 5.72 (t, *J* = 1.8 Hz, 1H), 5.70 (d, *J* = 1.7 Hz, 1H), 4.68 (dd, *J* = 5.1, 2.4 Hz, 1H), 4.18 (qd, *J* = 6.7, 2.5 Hz, 1H), 3.90–3.71 (m, 2H), 2.49 (d, *J* = 6.7 Hz, 1H), 2.41 (s, 3H), 1.23 (s, 9H), 1.04 (s, 9H); ^13^C{^1^H} NMR (100 MHz, CDCl_3_) δ 165.8, 165.4, 149.7, 137.2, 135.6, 135.6, 133.6, 133.6, 133.2, 133.1, 132.4, 130.4, 130.1, 130.1, 130.0, 129.9, 129.8, 129.3, 129.1, 128.6, 128.6, 127.8, 125.2, 91.5, 82.3, 82.2, 78.2, 77.3, 71.1, 65.0, 34.5, 31.4, 26.9, 20.6, 19.3; ESI HR-MS *m*/*z* [M + Na]^+^ calcd. for C_47_H_52_NaO_7_SSi: 811.3101, found 811.3105.

#### (2*R*,3*R*,4*R*,5*S*)-2-((*R*)-2-((((9*H*-Fluoren-9-yl)methoxy)carbonyl)oxy)-1-(benzoyloxy)ethyl)-5-((5-(*tert*-butyl)-2-methylphenyl)thio)tetrahydrofuran-3,4-diyl dibenzoate (**6**)

To a solution of **11b** (3.0 g, 3.80 mmol) in pyridine (30 mL) was added PhCOCl (0.66 mL, 5.70 mmol) dropwise at 0 °C, and the resulting mixture was gradually warmed to room temperature. The reaction mixture was stirred for 4 h at the same temperature, at the end of which time TLC indicated it was finished. The reaction was quenched with MeOH, diluted with CH_2_Cl_2_, and the mixture was washed with 1 M HCl, aq. NaHCO_3_, brine and dried over MgSO_4_. The combined organic layers were filtered, and concentrated. The residue was purified by silica gel column chromatography (ethyl acetate/*n*-hexanes: 20/80) to afford corresponding 2,3,5-*O*-benzoylated derivative in 78% yield (2.64 g) as a light-brown sticky liquid.

To a stirred solution of 2,3,5-*O*-benzoylated derivative (2.64 g, 2.95 mmol) in anhydrous THF/Py at 0 °C, 30% HF·Py (2.66 mL, 29.55 mmol) was added and stirred for 3.5 h. After completion of the reaction as indicated by TLC, the reaction was diluted with ethyl acetate, aq. NaHCO_3_ was added to quench the excess of acid and the two layers were separated. The organic portion was washed with 1 M HCl, aq. NaHCO_3_, brine. The combined organic layers were dried over MgSO_4_, concentrated and purified (ethyl acetate/*n*-hexanes: 60/40) to afford corresponding 6-*O*-alcohol in 71% yield (1.37 g) as a light yellow liquid.

The 6-*O*-alcohol (1.37 g, 2.09 mmol) from above step was dissolved in anhydrous CH_2_Cl_2_ at 0 °C, FmocCl (1.35 g, 5.23 mmol) and pyridine (0.84 mL, 10.46 mmol) were successively added and stirred at same temperature under ice bath for 4 h. After completion of the reaction as indicated by TLC, the reaction mixture was diluted with CH_2_Cl_2_ and washed with 1 M HCl, aq. NaHCO_3_, brine. The combined organic layers were dried over MgSO_4_, concentrated and purified by column chromatography using silica gel (ethyl acetate/*n*-hexanes: 20/80) to give **6** in 80% yield (1.46 g) as a white foam: ^1^H NMR (400 MHz, CDCl_3_) δ 8.10 (ddtd, *J* = 14.0, 7.5, 1.3, 0.6 Hz, 4H), 7.93–7.89 (m, 2H), 7.74 (dt, *J* = 7.6, 0.8 Hz, 2H), 7.66 (t, *J* = 1.7 Hz, 1H), 7.64–7.57 (m, 1H), 7.54 (dddt, *J* = 6.7, 3.8, 2.1, 0.8 Hz, 3H), 7.52–7.43 (m, 3H), 7.42–7.32 (m, 3H), 7.32–7.29 (m, 1H), 7.28 (dt, *J* = 2.4, 1.0 Hz, 1H), 7.26 (d, *J* = 0.4 Hz, 2H), 7.25–7.21 (m, 2H), 7.15 (ddd, *J* = 8.0, 1.1, 0.6 Hz, 1H), 6.09–6.01 (m, 1H), 5.78 (dq, *J* = 1.5, 0.8 Hz, 1H), 5.71 (td, *J* = 1.6, 1.0 Hz, 1H), 5.65 (ddq, *J* = 4.0, 1.5, 0.8 Hz, 1H), 4.92 (tdd, *J* = 4.0, 1.7, 0.8 Hz, 1H), 4.73–4.52 (m, 2H), 4.43–4.31 (m, 1H), 4.30–4.08 (m, 2H), 2.46 (s, 3H), 1.26 (s, 9H); ^13^C{^1^H} NMR (100 MHz, CDCl_3_) δ 165.8, 165.6, 165.4, 154.9, 149.9, 143.4, 143.3, 141.3, 137.3, 133.7, 133.5, 133.4, 132.1, 130.6, 130.2, 130.0, 129.4, 129.0, 128.9, 128.6, 128.5, 127.9, 127.9, 127.3, 127.2, 125.4, 125.4, 125.3, 120.0, 91.5, 82.6, 81.5, 77.9, 77.3, 70.3, 70.2, 66.3, 46.6, 34.5, 31.4, 20.5; ESI HR-MS *m*/*z* [M + Na]^+^ calcd. for C_53_H_48_NaO_10_S: 899.2866, found 899.2869.

#### Analytical Data for 20-mer Oligogalactofuranoside (**14**)

Sticky solid (Yield: 17 mg, 13% over 41 steps). ^1^H NMR (700 MHz, CDCl_3_) δ 8.04–7.80 (m, 85H), 7.76–7.51 (m, 45H), 7.49–7.43 (m, 14H), 7.42–7.30 (m, 52H), 7.23 (t, *J* = 7.6 Hz, 12H), 7.19–7.01 (m, 75H), 6.99–6.87 (m, 22H), 5.85–5.55 (m, 57H), 4.88–4.23 (m, 85H), 1.46 (m, 4H), 1.37–1.32 (m, 4H); ^13^C{^1^H} NMR (175 MHz, CDCl_3_) δ 165.9, 165.6, 165.3, 133.4, 133.0, 132.8, 129.9, 129.8, 129.8, 129.7, 129.1, 128.7, 128.6, 128.3, 128.0, 105.4, 83.6, 81.9, 72.9, 65.4, 32.0, 29.8, 29.5, 29.1, 23.4, 22.8, 14.2; (MALDI-TOF) *m*/*z* [M + K]^+^ calcd. for C_553_H_459_KNO_163_: 9758.7296, found 9758.5120.

#### 20-mer Oligogalactofuranoside (**1**)

Sodium methoxide in methanol (0.5 M, *p*H = 13) was added to a solution of protected oligosaccharide (17 mg) **14** in methanol:CH_2_Cl_2_ (1:1), and stirred at room temperature for 16 h, neutralized with Amberlite ion exchange (H^+^) resin, filtered and concentrated in vacuo and carried forward directly into hydrogenolysis without purification. The Zemplén methanolysis product was dissolved in EtOAc:*t*-BuOH:H_2_O (2:1:1) and transferred to cylindrical vials. Pd(OH)_2_/C (10%), (100 wt %) was added and the reaction mixture was stirred in hydrogen reactor with 5 bar pressure for 4 h. The reaction mixture was filtered through a pad of Celite and washed with methanol and water. The filtrates were concentrated in vacuo and purified on size exclusion chromatography (Method B) Synergi Hydro RP18 column and lyophilized to give a pure compound **1** in 40% yield over 2 steps (2 mg) as a white fluffy solid. Analytical data for **1**: ^1^H NMR (700 MHz, D_2_O) δ 5.38–5.13 (m, 17H), 5.08–4.97 (m, 3H), 4.34 (d, *J* = 3.4 Hz, 2H), 4.21–4.12 (m, 48H), 4.11–4.06 (m, 8H), 4.03–3.96 (m, 17H), 3.92 (ddd, *J* = 14.3, 8.5, 3.2 Hz, 4H), 3.83 (t, *J* = 4.8 Hz, 33H), 3.79–3.59 (m, 10H), 3.09–3.00 (m, 2H), 1.80–1.63 (m, 4H), 1.53–1.43 (m, 2H); ^13^C{^1^H} NMR (175 MHz, D_2_O) δ 107.2, 107.1, 107.0, 107.0, 106.9, 82.6, 82.5, 82.4, 81.4, 81.4, 81.3, 81.2, 81.0, 79.5, 76.6, 76.4, 76.0, 75.5, 70.6, 70.5, 68.1, 62.8, 62.8, 61.1, 60.9, 39.4, 28.1, 26.4, 22.2; (MALDI-TOF) *m*/*z* [M + Na]^+^ calcd. for C_125_H_213_NNaO_101_: 3367.1460, found 3367.1450.

## Supporting Information Available

Copies of ^1^H NMR, ^13^C NMR, HPLC chromatograms, and MALDI (PDF)
