# Supplementary material for: Automated Glycan Assembly of Oligogalactofuranosides Reveals the Influence of Protecting Groups on Oligosaccharide Stability
Source: J Org Chem. 2021 May 7;86(10):7280–7. doi: 10.1021/acs.joc.1c00505 (PMC8154612; doi:10.1021/acs.joc.1c00505)
Supplement: Supplementary file 1 — jo1c00505_si_001.pdf [file jo1c00505_si_001.pdf]

Supporting Information

Automated Glycan Assembly of Oligogalactofuranosides Reveals the Influence of Protecting Groups on Oligosaccharide Stability

Narayana Murthy Sabbavarapu,[†] Peter H. Seeberger*,[†,‡]

[†]Department of Biomolecular Systems, Max-Planck-Institute of Colloids and Interfaces, 14476 Potsdam, Germany

[‡]Freie Universität Berlin, Institute of Chemistry and Biochemistry, 14195 Berlin, Germany

Email: peter.seeberger@mpikg.mpg.de

Table of Contents

1. Automated Glycan Assembly of Oligogalactofuranosides ……………………………..S3
   1.1 Synthesis of *β*-(1→5) and *β*-(1→6)-linked linear galactan heptamer **10** using building blocks **3** and **4**………………………………………………………S3
   1.2 Synthesis of β-(1→5) and β-(1→6)-linked linear galactan heptamer **13** using building blocks **5** and **6**……………………………………………………….S4
   1.3 Synthesis of *β*-(1→5) and *β*-(1→6)-linked linear galactan 20-mer **14** using building blocks **5** and **6**…………………………………………………………S6
2. Copies of NMR Spectra…………………………………………………S9-S35

1. Automated Glycan Assembly of Oligogalactofuranosides

1.1 Synthesis of *β*-(1→5) and *β*-(1→6)-linked linear galactan heptamer 10 using building blocks 3 and 4

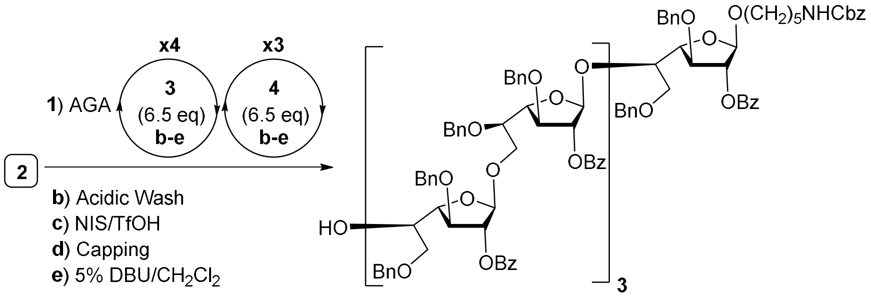

10

Module	Conditions	Cycles
A: Resin Preparation for Synthesis		
B: Acidic Wash with TMSOTf Solution<br>C: Thioglycoside Glycosylation<br>D: Capping<br>E: Fmoc Deprotection	Building block **3** (6 equiv)<br>-20° for 15 min, 0° for 20 min	4
B: Acidic Wash with TMSOTf Solution<br>C: Thioglycoside Glycosylation<br>D: Capping<br>E: Fmoc Deprotection	Building block **4** (6 equiv)<br>-20° for 15 min, 0° for 20 min	3

The product was cleaved from the solid support as described in the post-synthesizer manipulations followed by purification using normal phase preparative HPLC with a (YMC-Diol-300 column, 150 x 4.6 mm) flow rate of 1.0 mL / min with Hex – 20% EtOAc as eluents [isocratic 20% EtOAc (5 min), linear gradient to 60% EtOAc (5 min), linear gradient to 60% EtOAc (30 min), linear gradient to 100% EtOAc (5 min)].

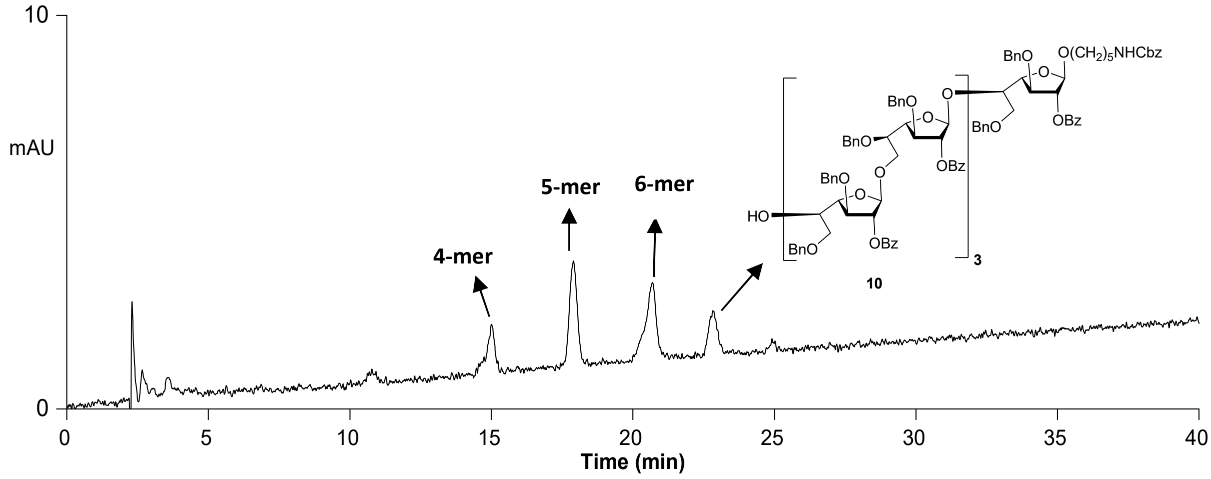

Figure S1. Analytical HPLC (Method A, 280 nm) of crude protected galactan 7-mer **10**

1.2 Synthesis of *β*-(1→5) and *β*-(1→6)-linked linear galactan heptamer 13 using building blocks 5 and 6

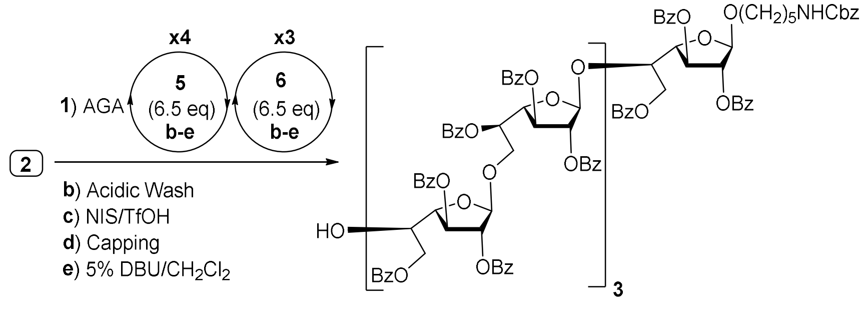

13

Module	Conditions	Cycles
A: Resin Preparation for Synthesis		
B: Acidic Wash with TMSOTf Solution		4
C: Thioglycoside Glycosylation	Building block **5** (6 equiv) -20º for 15 min, 0º for 20 min	
D: Capping		
E: Fmoc Deprotection		
B: Acidic Wash with TMSOTf Solution		3
C: Thioglycoside Glycosylation	Building block **6** (6 equiv) -20º for 15 min, 0º for 20 min	
D: Capping		
E: Fmoc Deprotection		

The product was cleaved from the solid support as described in the post-synthesizer manipulations followed by purification using normal phase preparative HPLC with a (YMC-Diol-300 column, 150 x 4.6 mm) flow rate of 1.0 mL / min with Hex – 20% EtOAc as eluents [isocratic 20% EtOAc (5 min), linear gradient to 60% EtOAc (5 min), linear gradient to 60% EtOAc (30 min), linear gradient to 100% EtOAc (5 min)].

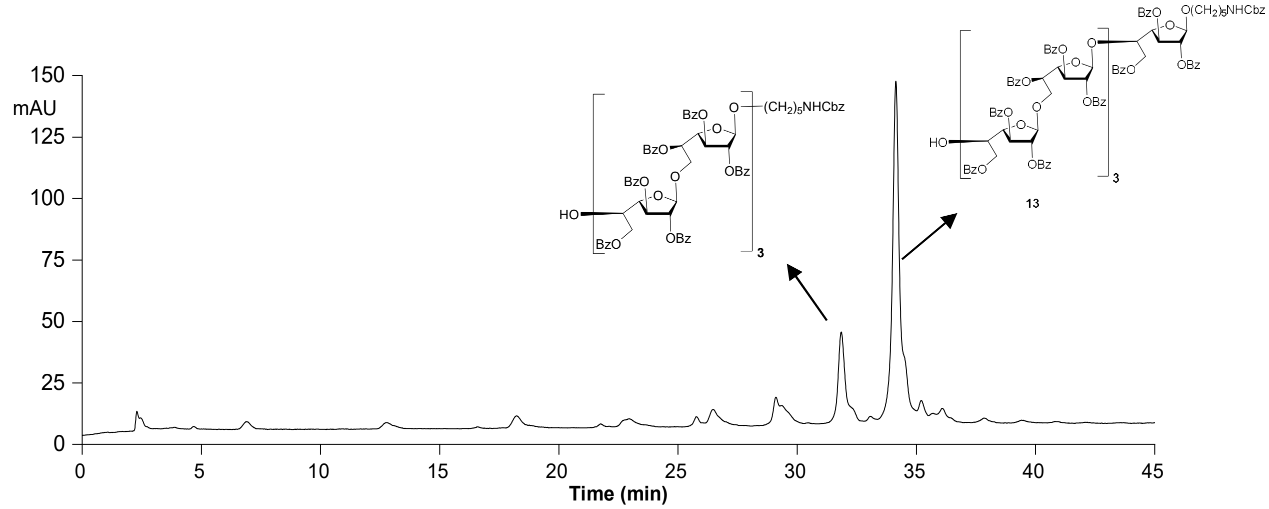

Figure S2. Analytical HPLC (Method A, 280 nm) of crude protected galactan 7-mer **13**

1.3 Synthesis of *β*-(1→5) and *β*-(1→6)-linked linear galactan 20-mer 14 using building blocks 5 and 6

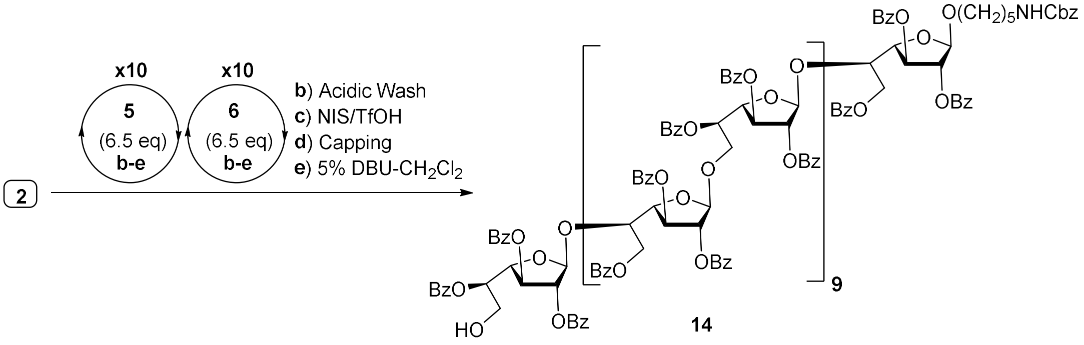

Module	Conditions	Cycles
A: Resin Preparation for Synthesis		
B: Acidic Wash with TMSOTf Solution<br>C: Thioglycoside Glycosylation<br>D: Capping<br>E: Fmoc Deprotection	Building block **5** (6 equiv)<br>-20° for 15 min, 0° for 20 min	10
B: Acidic Wash with TMSOTf Solution<br>C: Thioglycoside Glycosylation<br>D: Capping<br>E: Fmoc Deprotection	Building block **6** (6 equiv)<br>-20° for 15 min, 0° for 20 min	10

The product was cleaved from the solid support as described in the post-synthesizer manipulations followed by purification using normal phase preparative HPLC with a (YMC-Diol-300 column, 150 x 4.6 mm) flow rate of 1.0 mL / min with Hex – 20% EtOAc as eluents [isocratic 20% EtOAc (5 min), linear gradient to 60% EtOAc (5 min), linear gradient to 60% EtOAc (30 min), linear gradient to 100% EtOAc (5 min)].

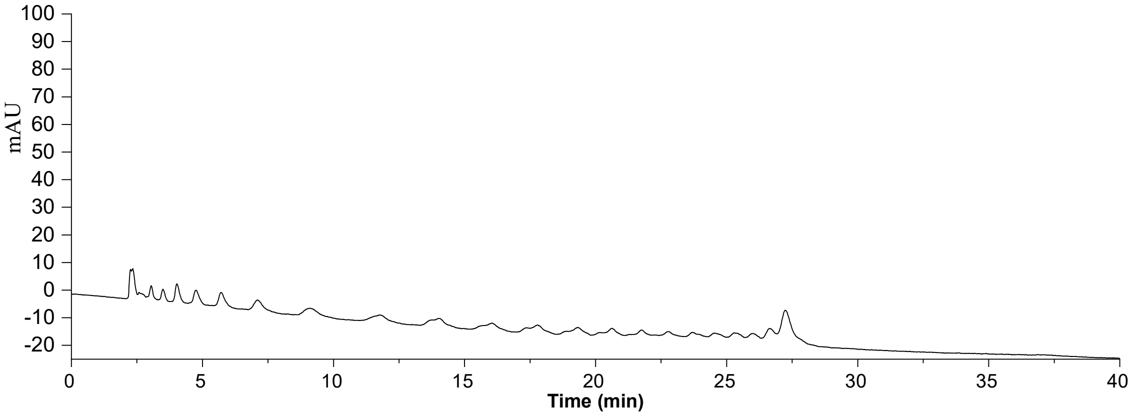

Figure S3. Analytical HPLC (Method A, 280 nm) of crude protected galactan 20-mer **14**

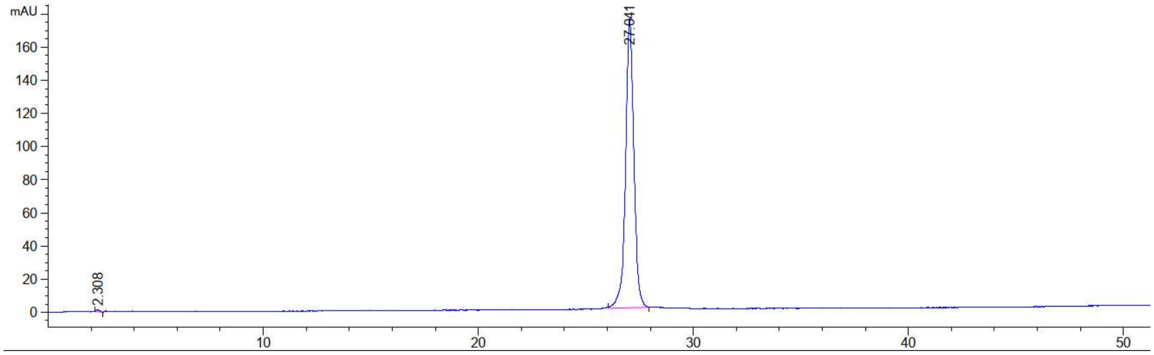

Figure S4. Analytical HPLC (Method A, 280 nm) of pure protected galactan 20-mer **14**

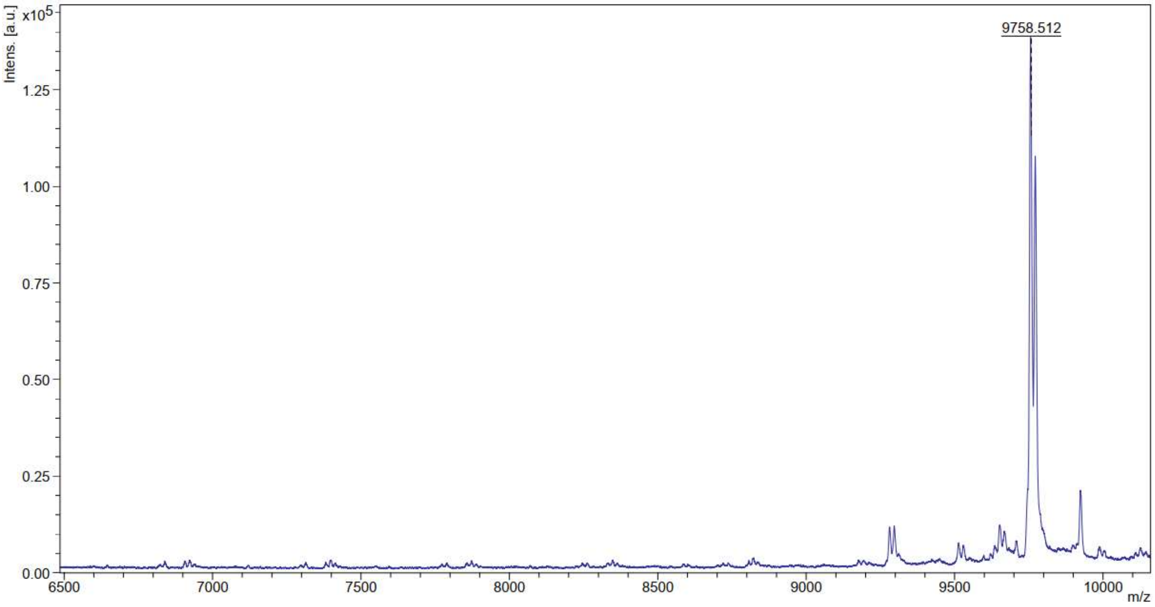

Figure S5. MALDI-TOF of pure protected galactan 20-mer **14**

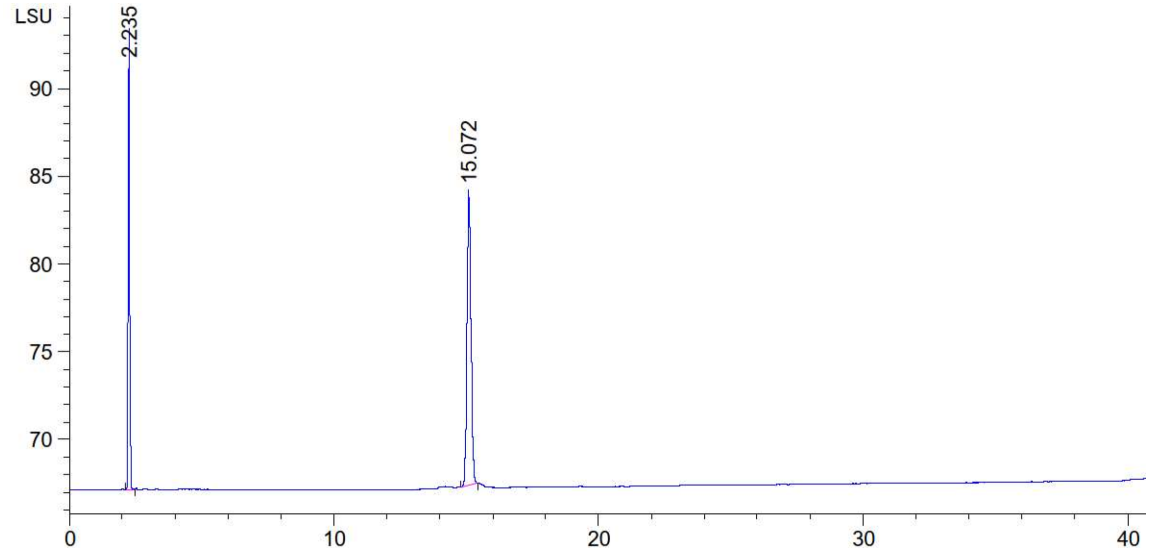

Figure S6. Analytical HPLC (Method B, ELSD trace) of pure galactan 20-mer **1**

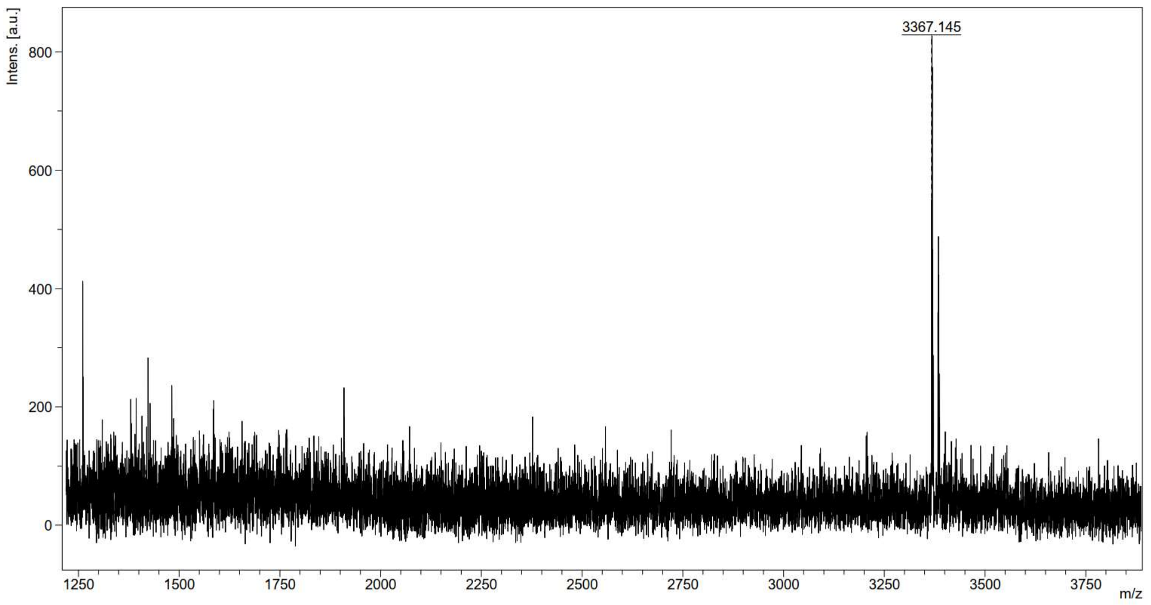

Figure S7. MALDI-TOF of pure galactan 20-mer **1**

2. Copies of NMR Spectra

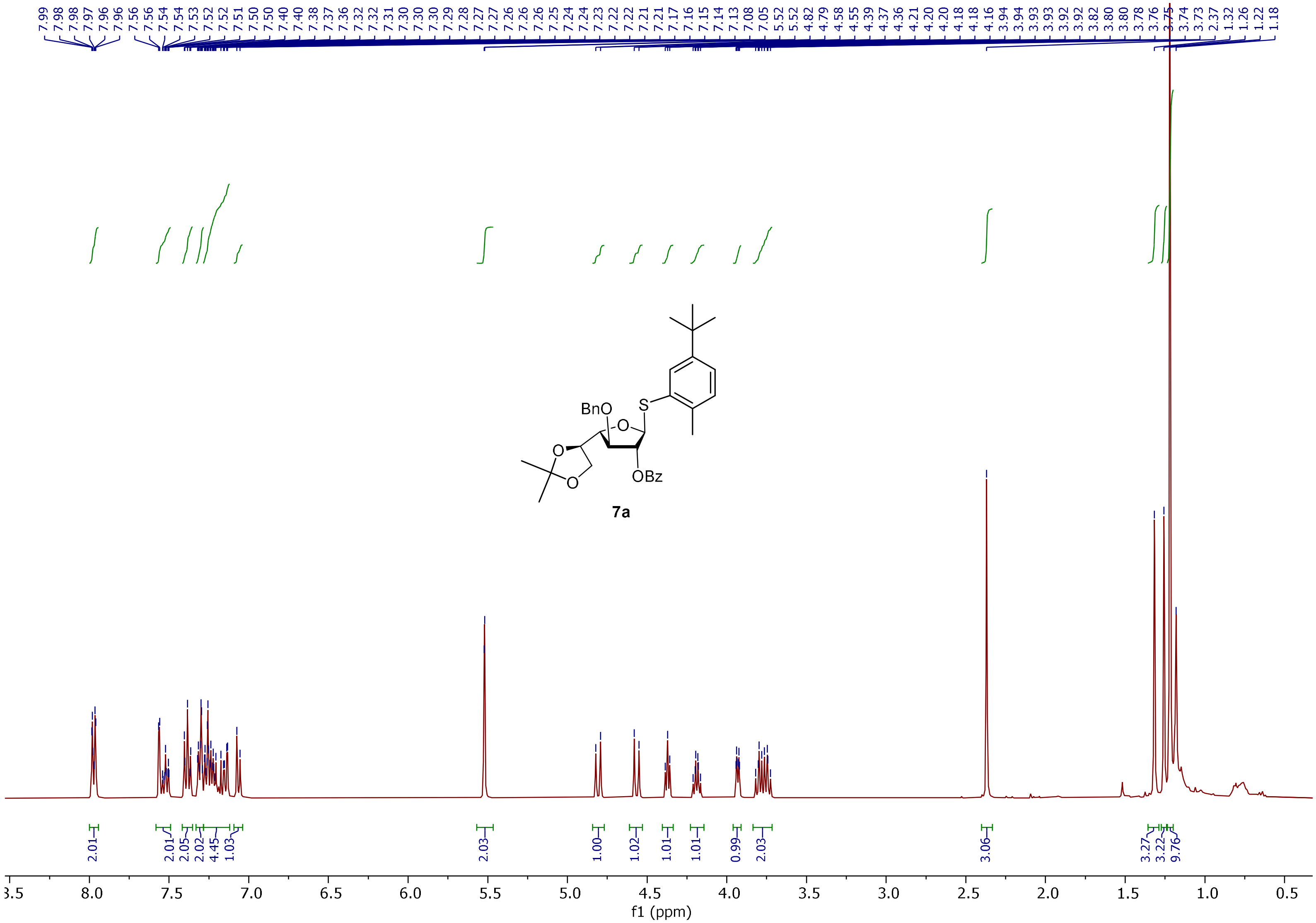

The [1]H Spectrum of Compound **7a** (400 MHz, $CDCl_3$)

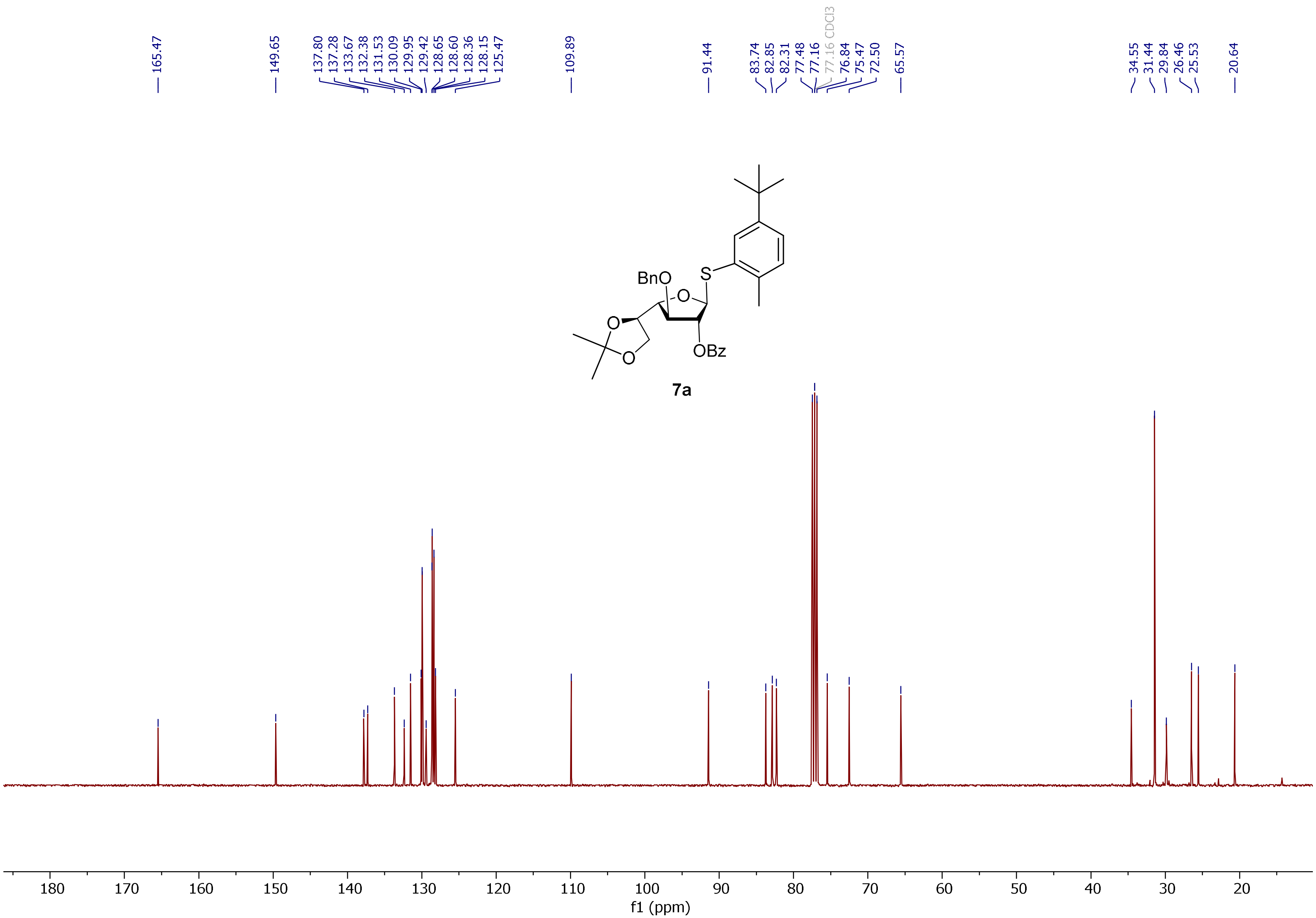

The $^{13}C\{^{1}H\}$ Spectrum of Compound **7a** (100 MHz, $CDCl_3$)

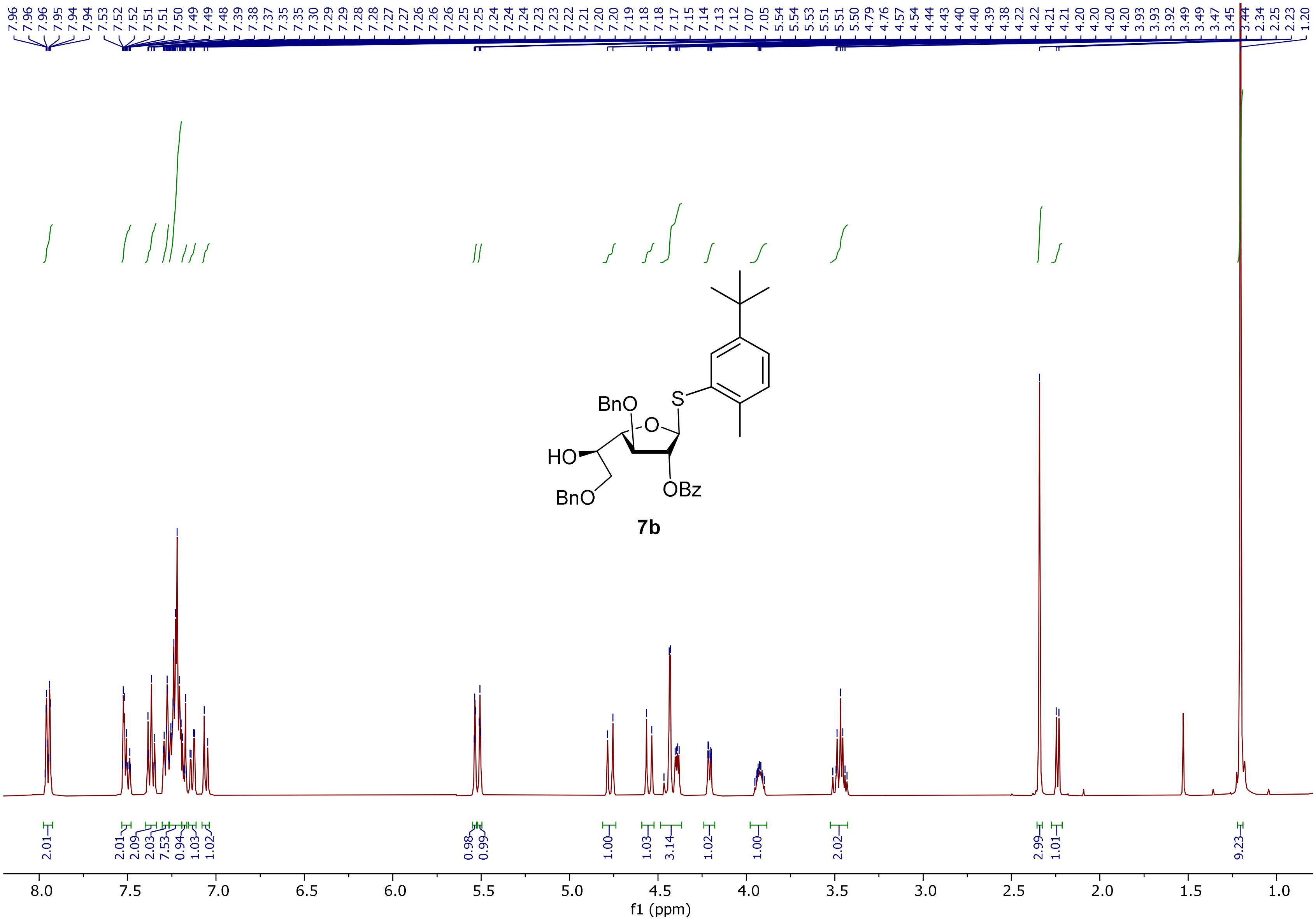

The ^{1}H Spectrum of Compound **7b** (400 MHz, $CDCl_3$)

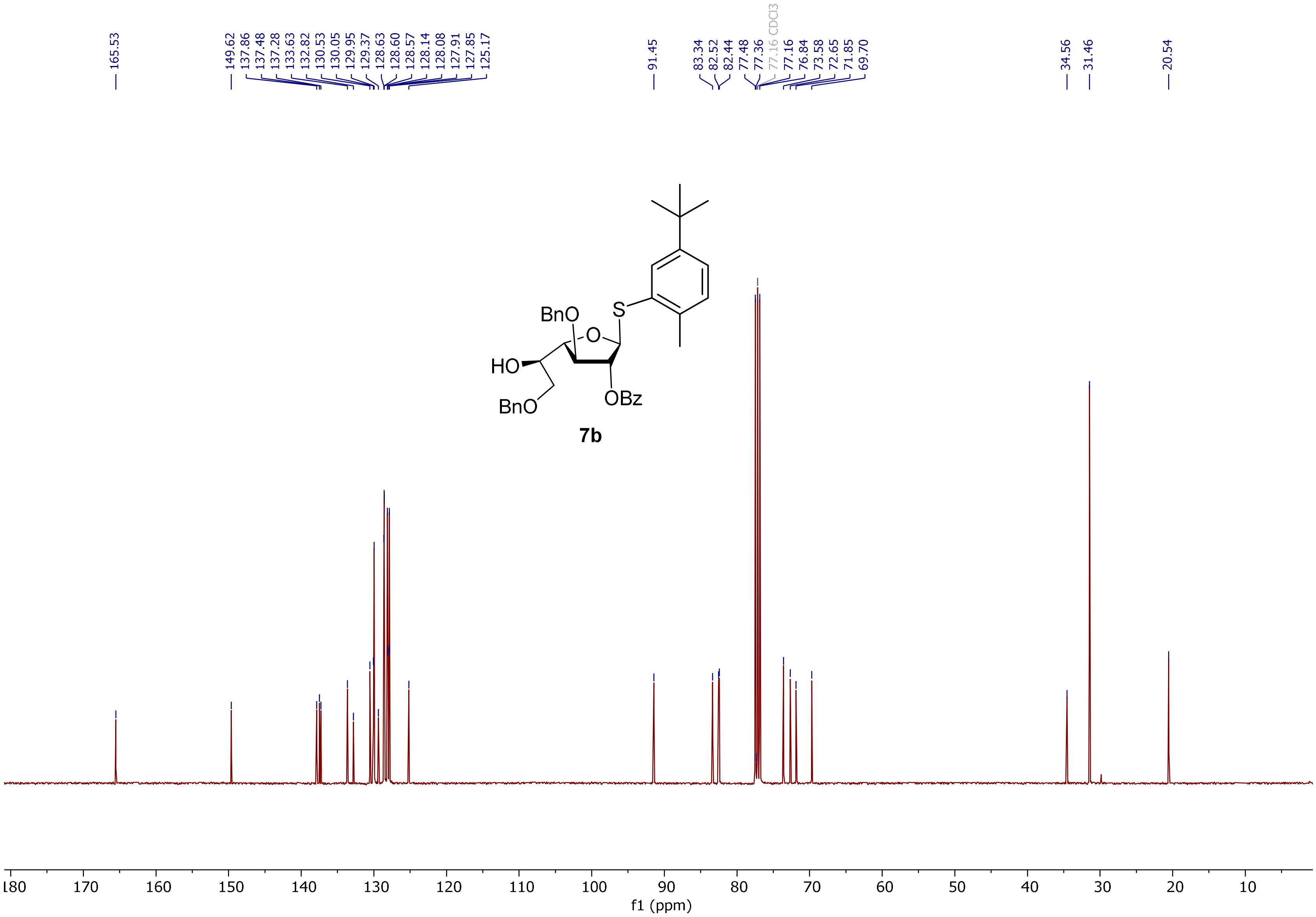

The $^{13}C\{^{1}H\}$ Spectrum of Compound **7b** (100 MHz, $CDCl_3$)

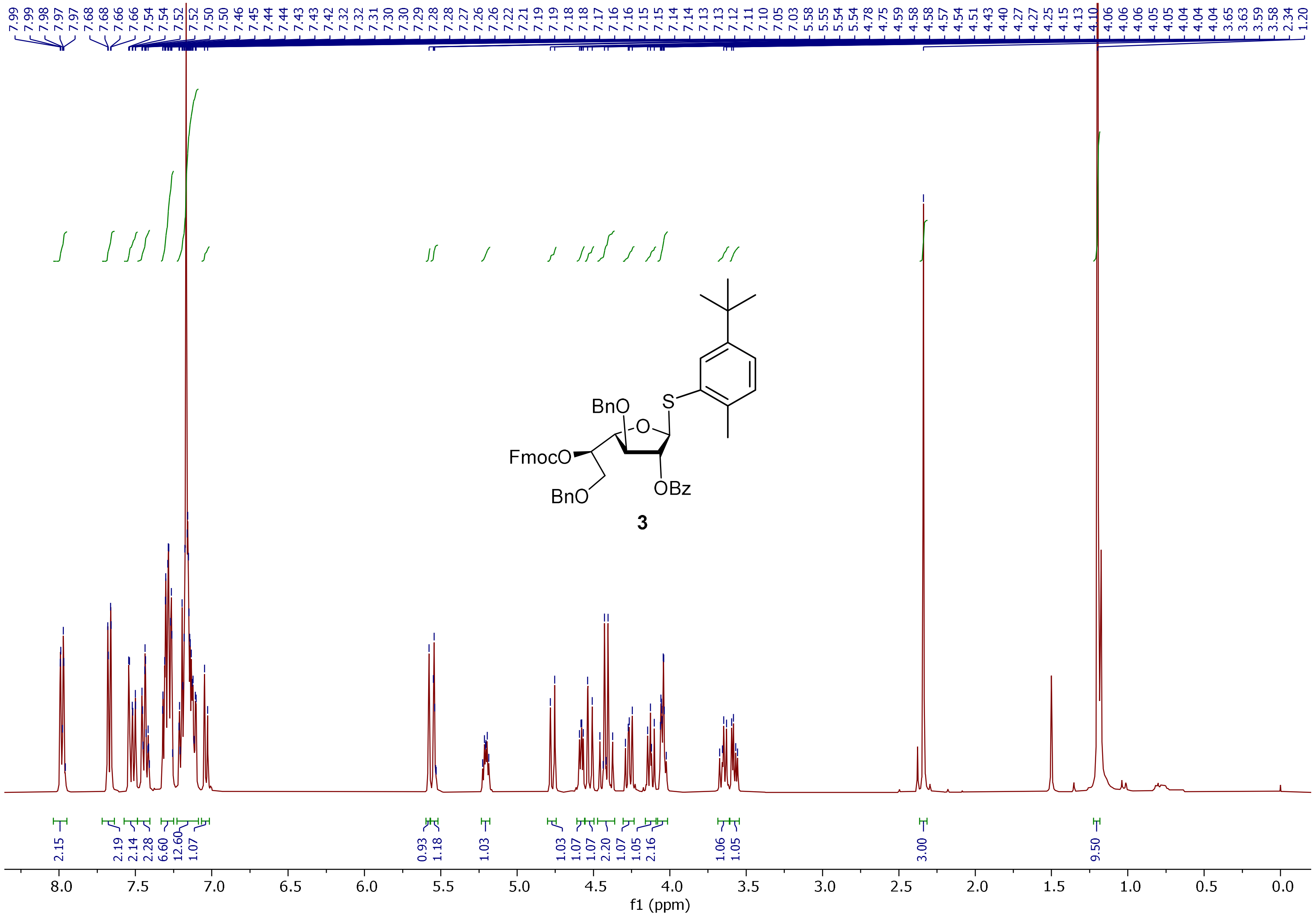

The [1]H Spectrum of Compound **3** (400 MHz, $CDCl_3$)

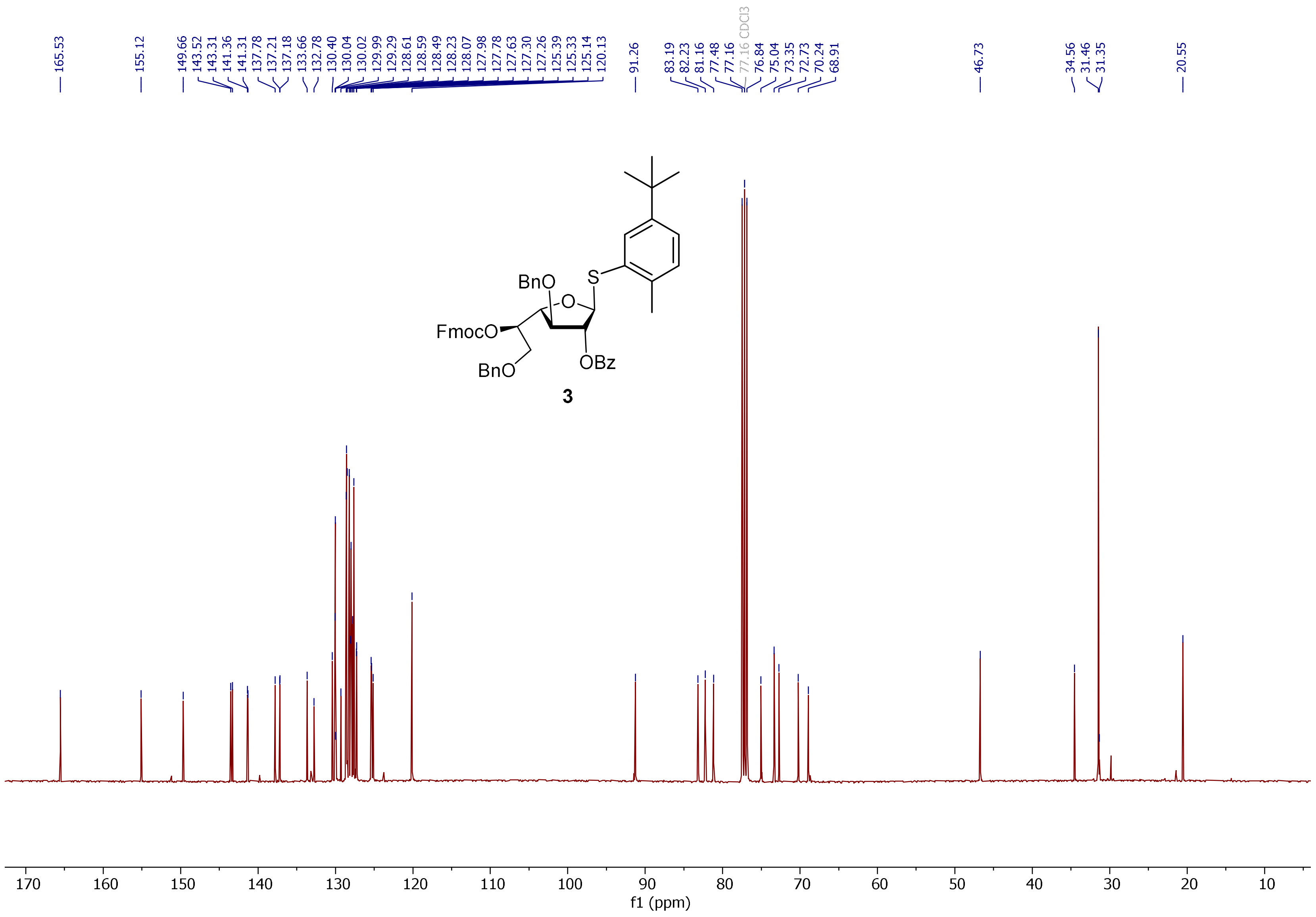

The $^{13}C\{^{1}H\}$ Spectrum of Compound **3** (100 MHz, $CDCl_3$)

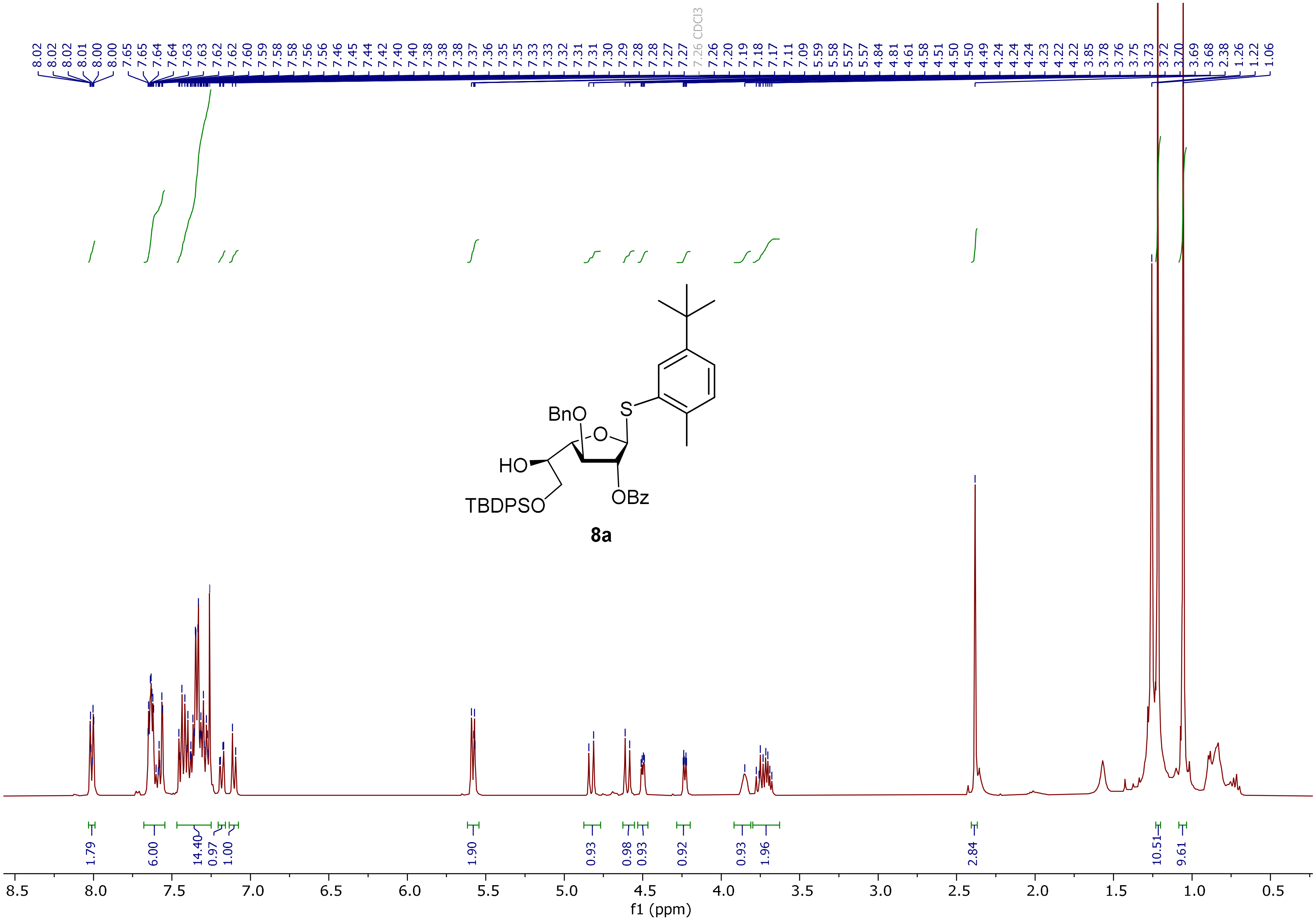

The [1]H Spectrum of Compound **8a** (400 MHz, $CDCl_3$)

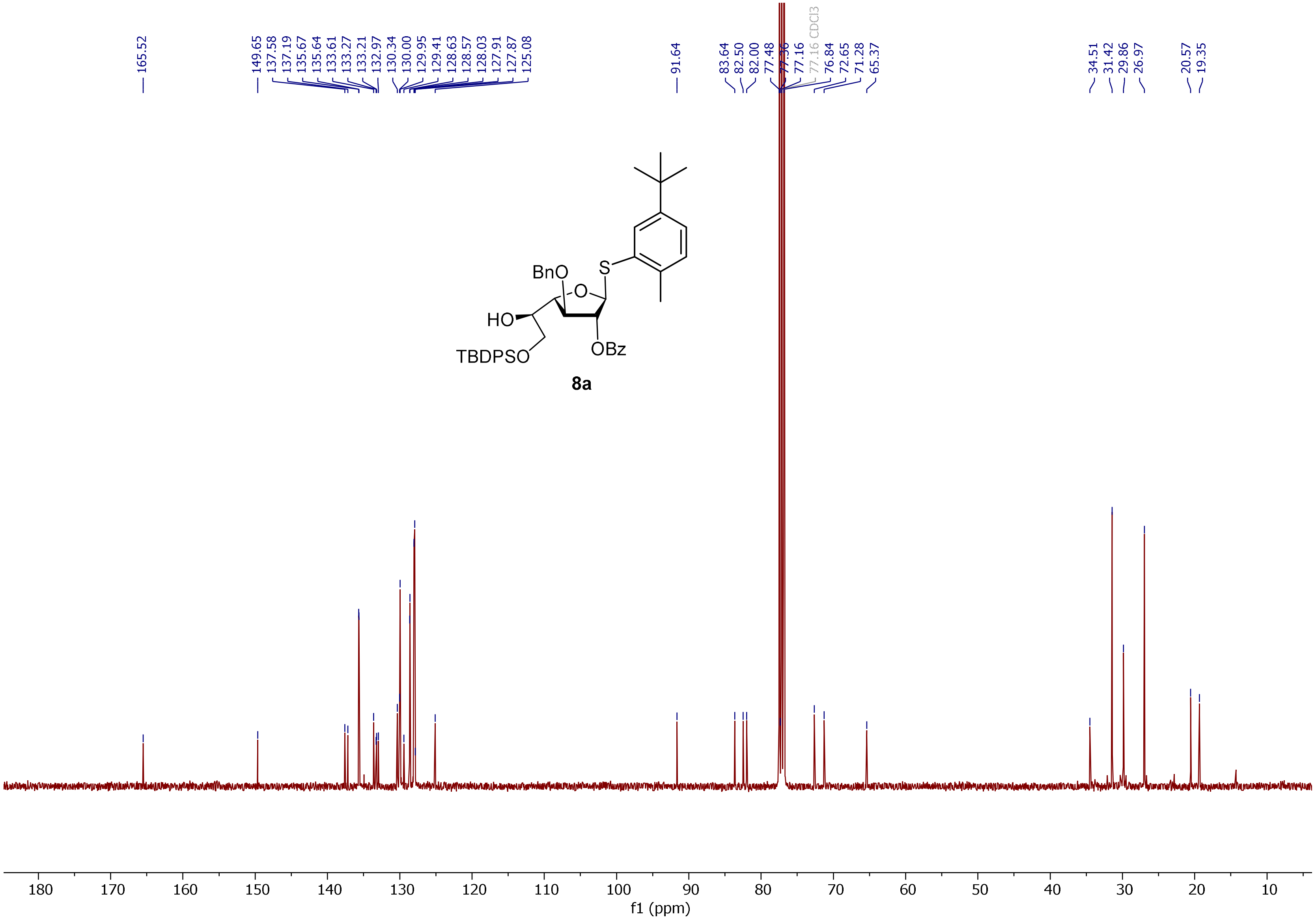

The $^{13}C\{^{1}H\}$ Spectrum of Compound **8a** (100 MHz, $CDCl_3$)

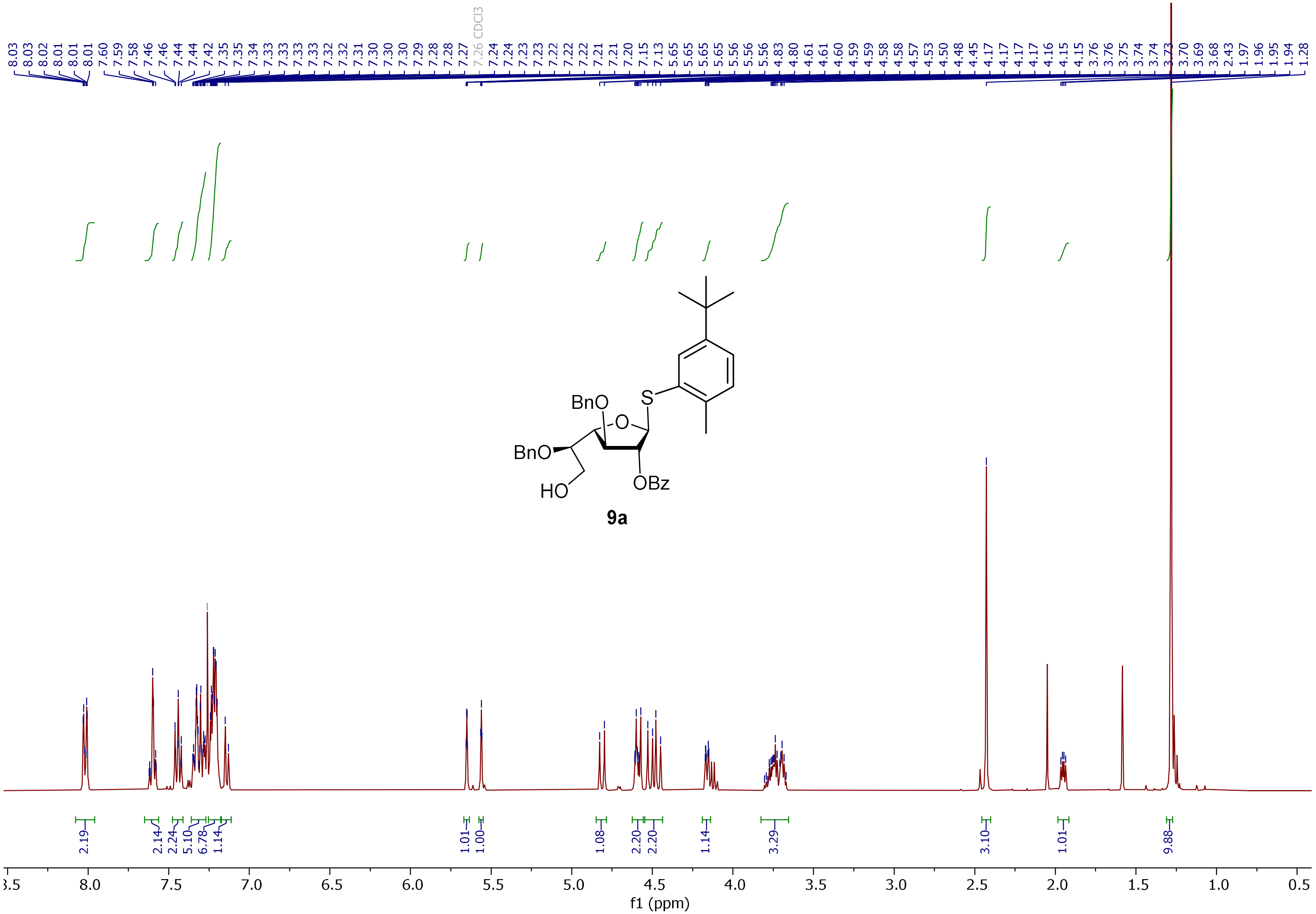

The [1]H Spectrum of Compound **9a** (400 MHz, $CDCl_3$)

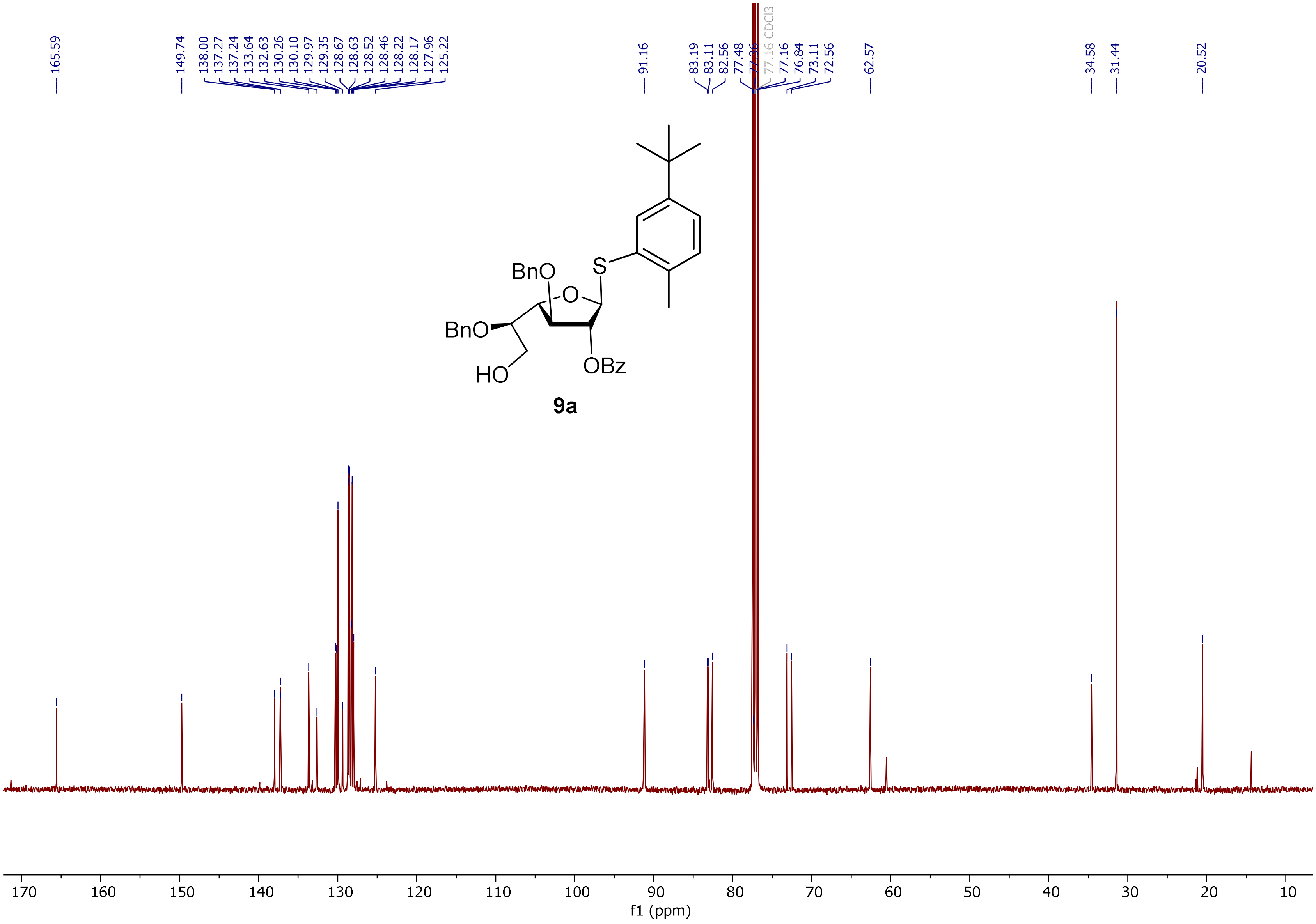

The $^{13}C\{^{1}H\}$ Spectrum of Compound **9a** (100 MHz, $CDCl_3$)

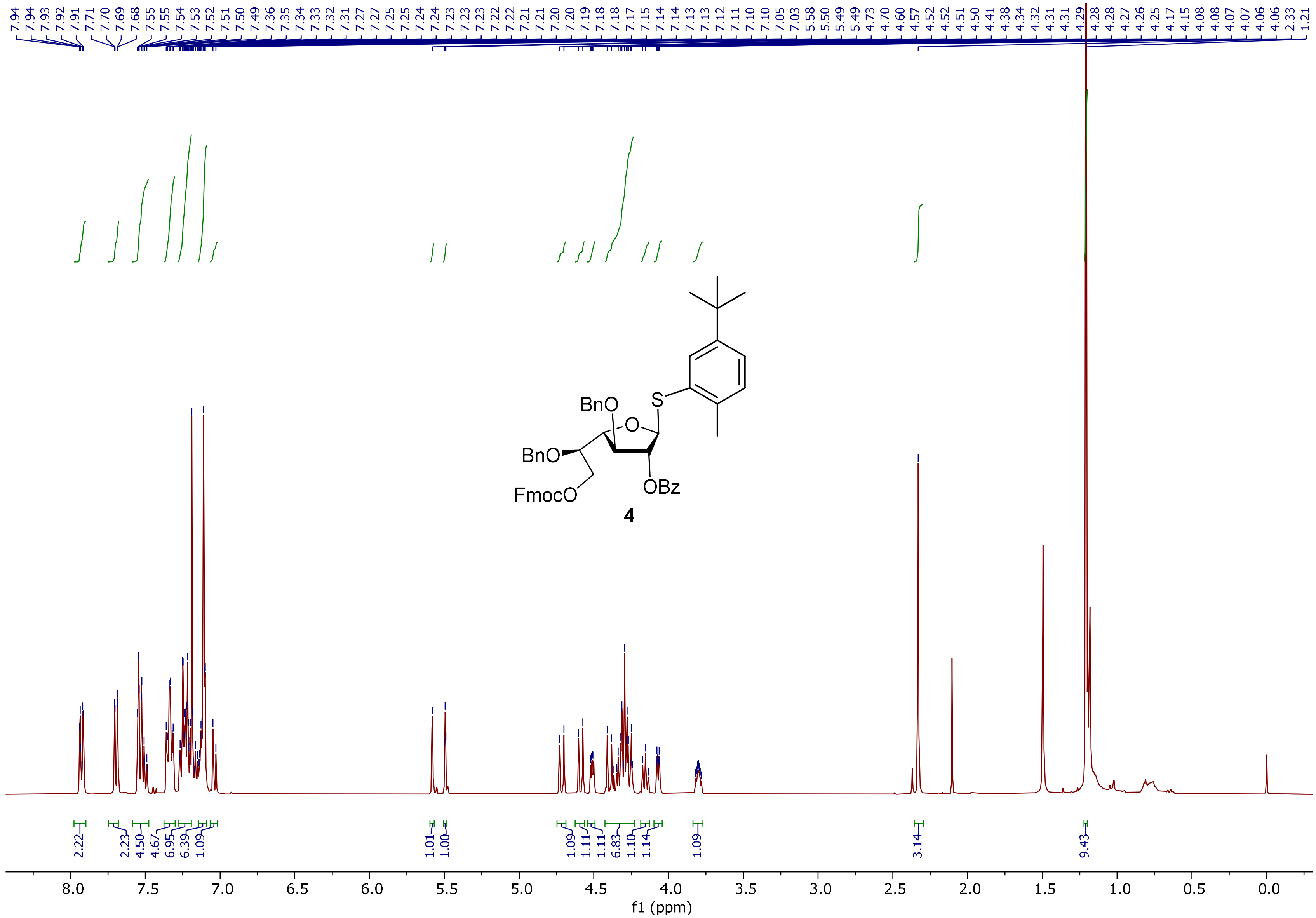

The [1]H Spectrum of Compound **4** (400 MHz, $CDCl_3$)

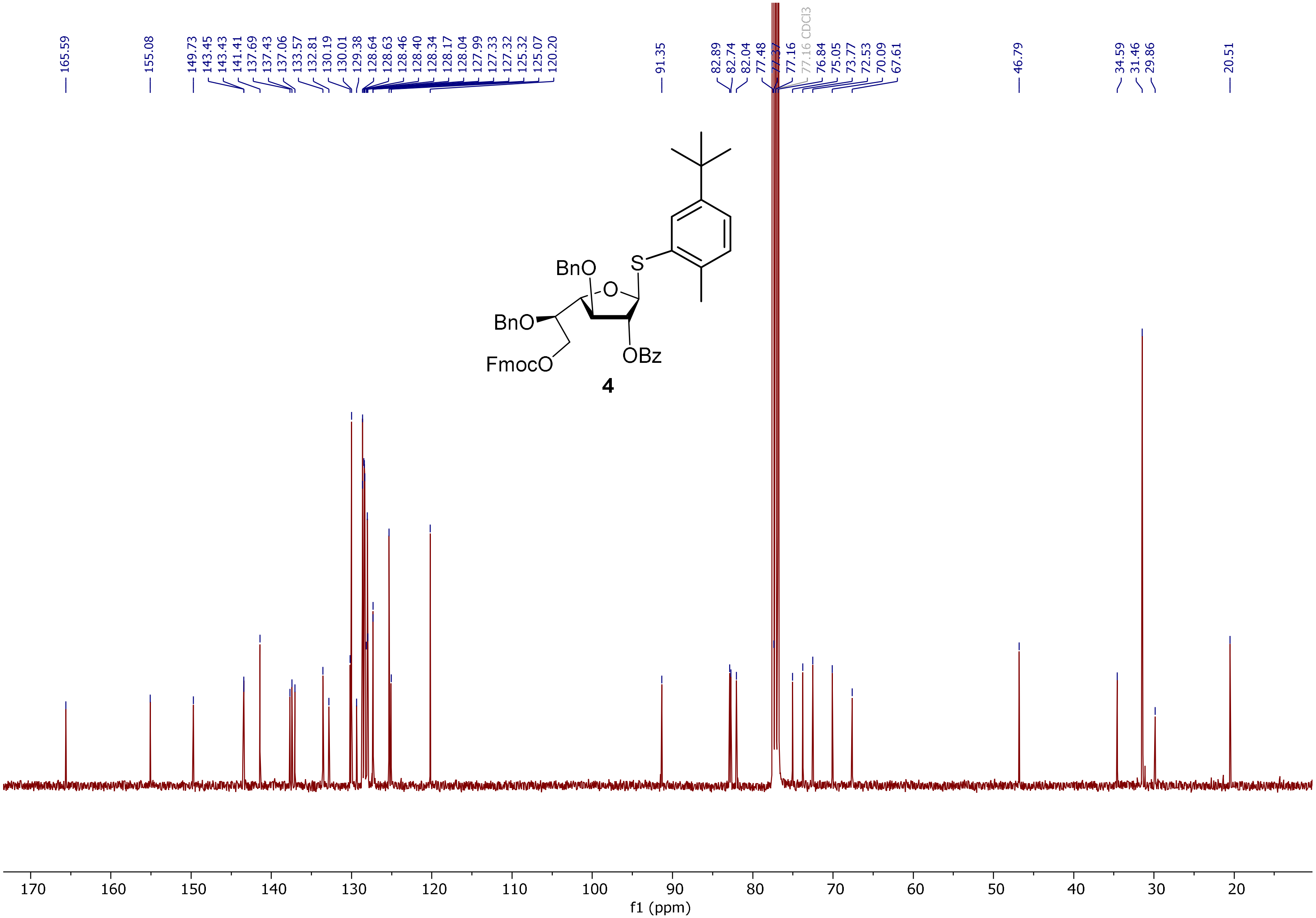

The $^{13}C\{^{1}H\}$ Spectrum of Compound **4** (100 MHz, $CDCl_3$)

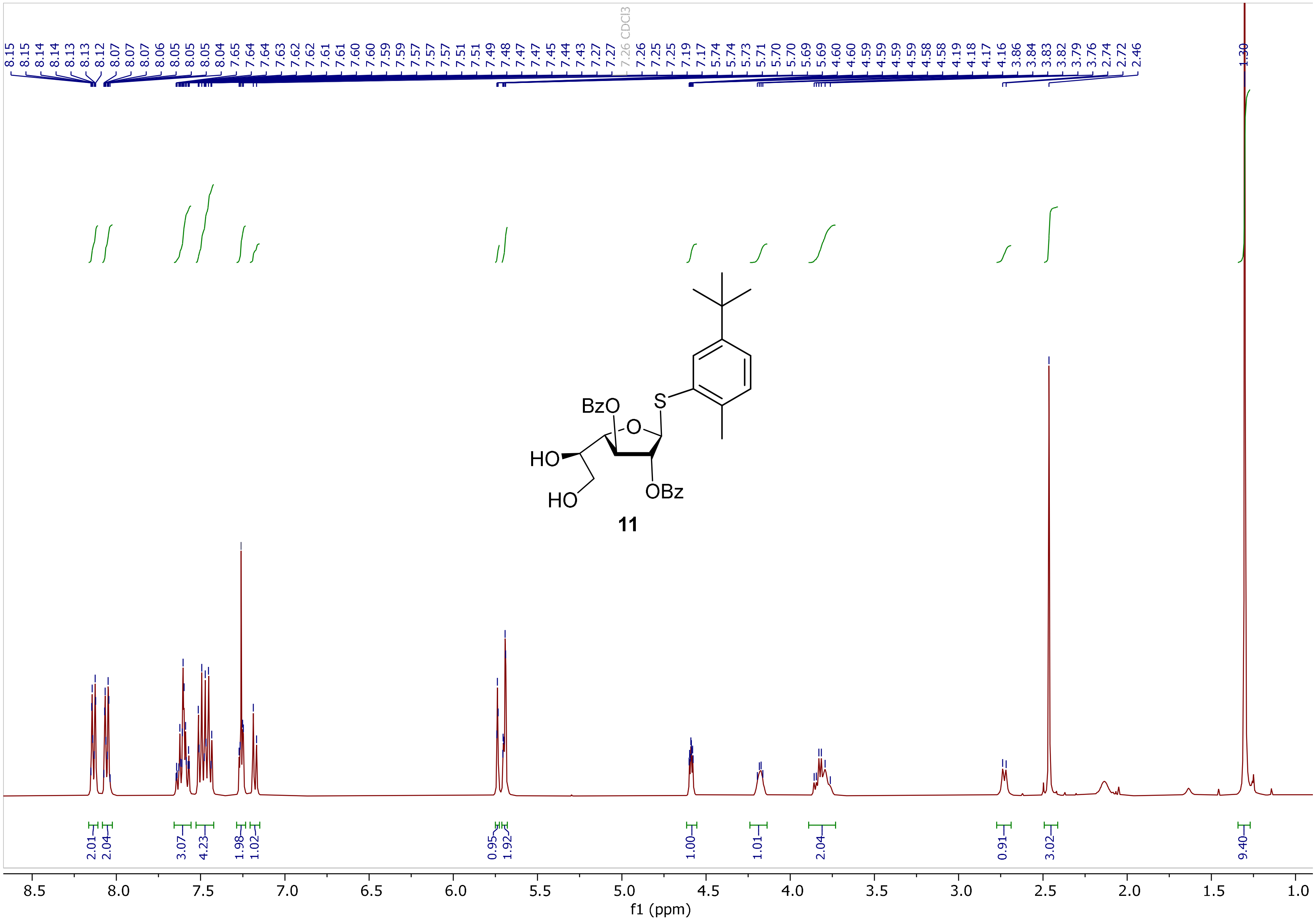

The ^{1}H Spectrum of Compound **11** (400 MHz, $CDCl_3$)

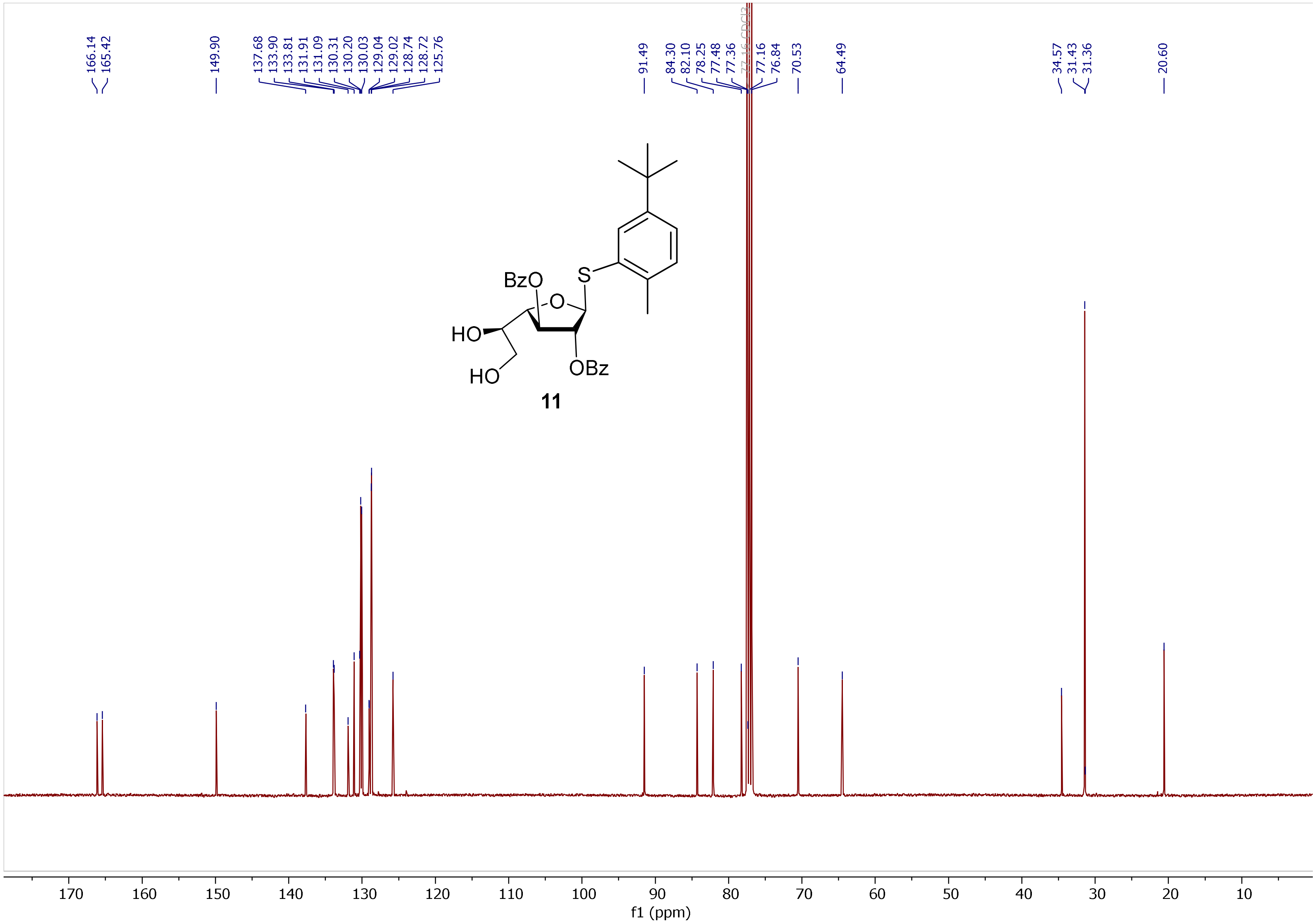

The $^{13}C\{^{1}H\}$ Spectrum of Compound **11** (100 MHz, $CDCl_3$)

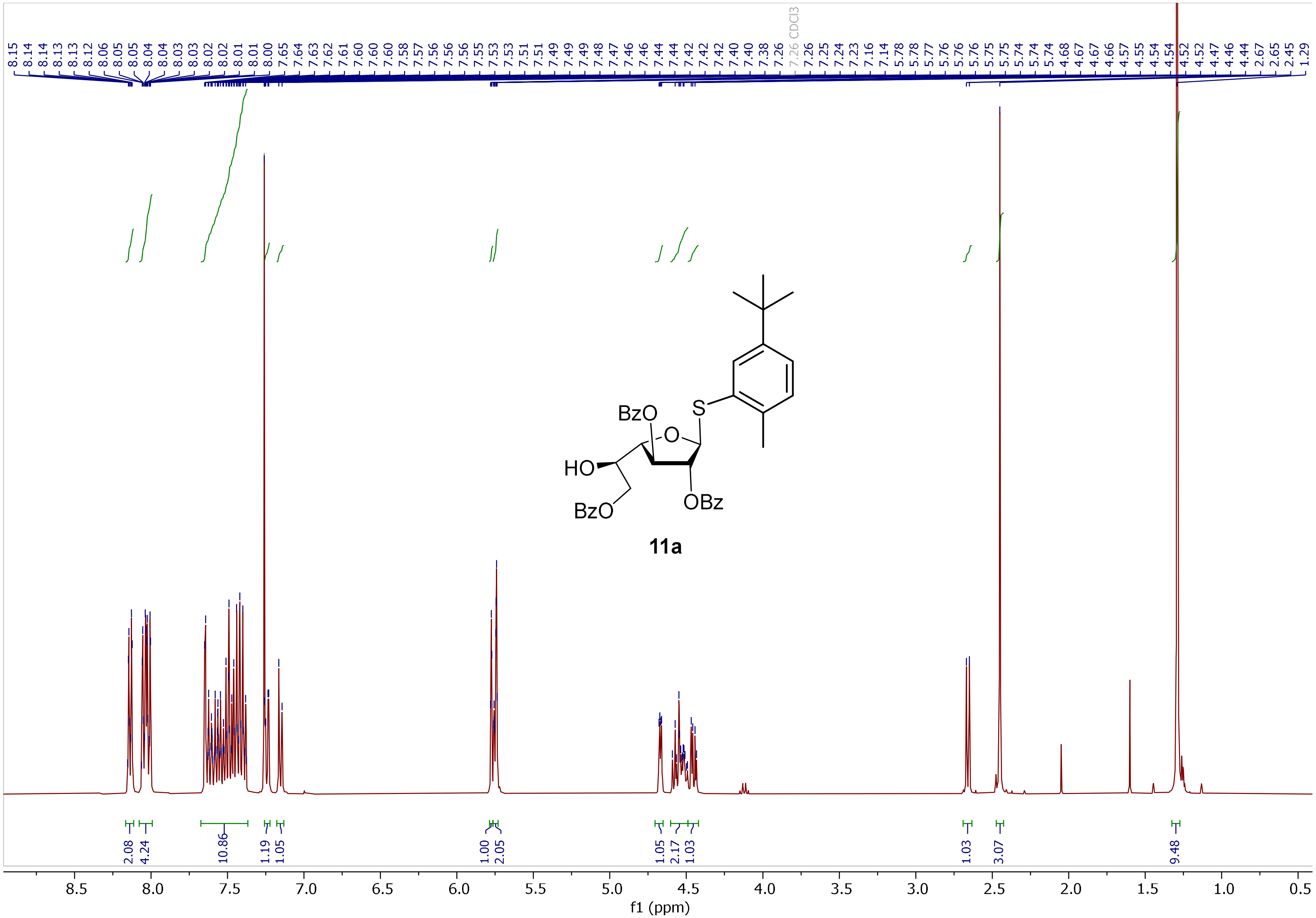

The ^{1}H Spectrum of Compound **11a** (400 MHz, $CDCl_3$)

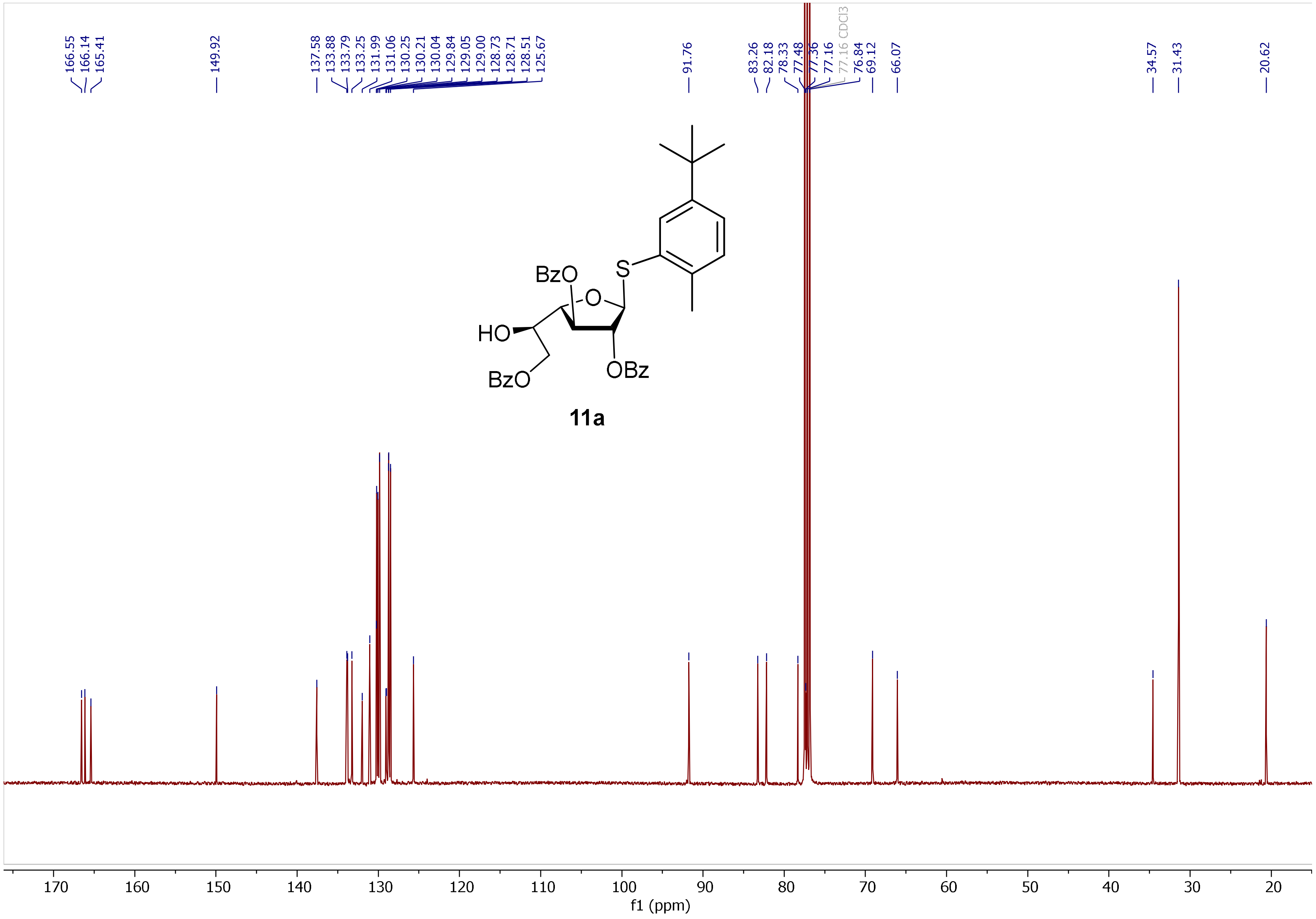

The $^{13}C\{^{1}H\}$ Spectrum of Compound **11a** (100 MHz, $CDCl_3$)

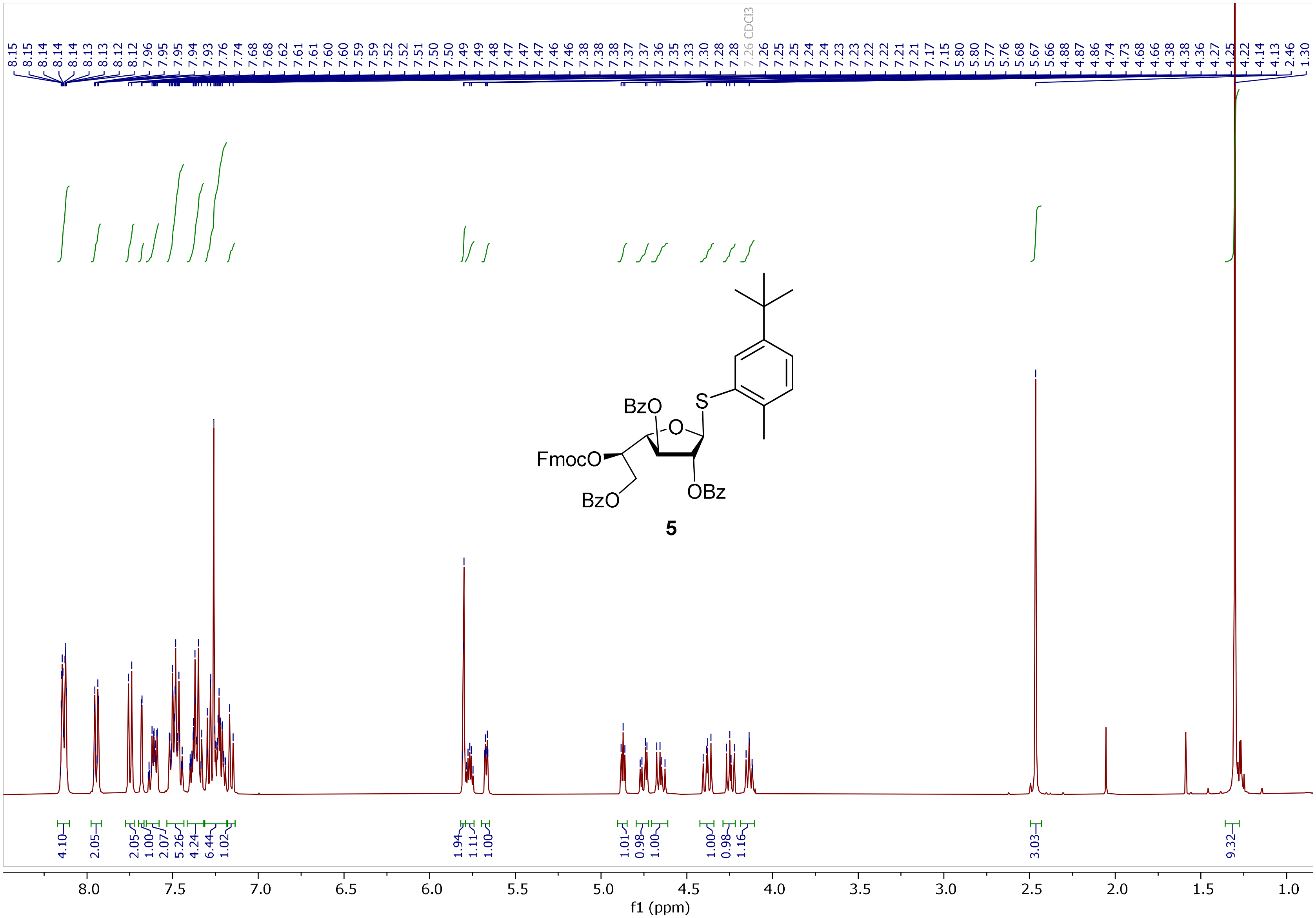

The [1]H Spectrum of Compound **5** (400 MHz, $CDCl_3$)

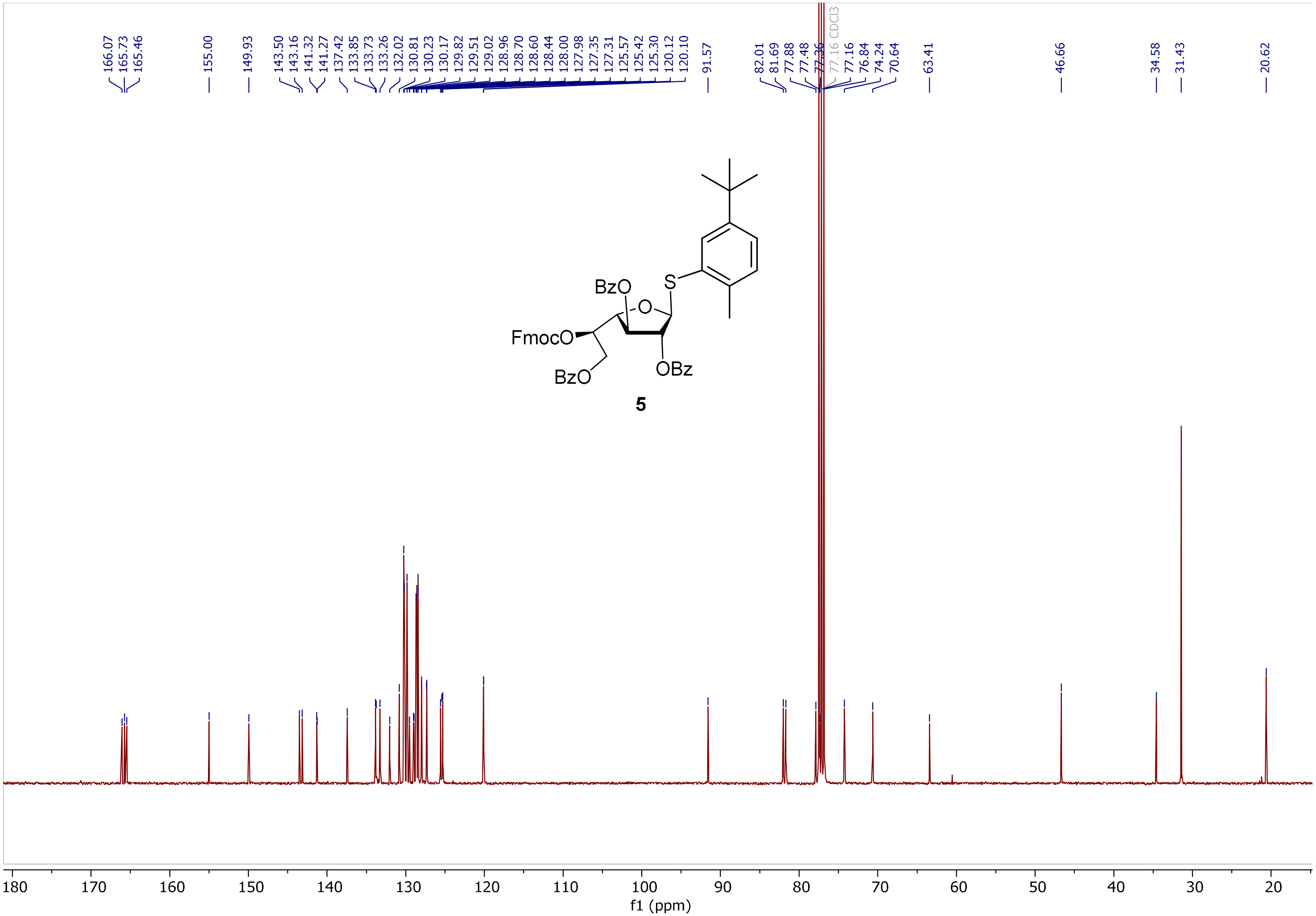

The $^{13}C\{^{1}H\}$ Spectrum of Compound **5** (100 MHz, $CDCl_3$)

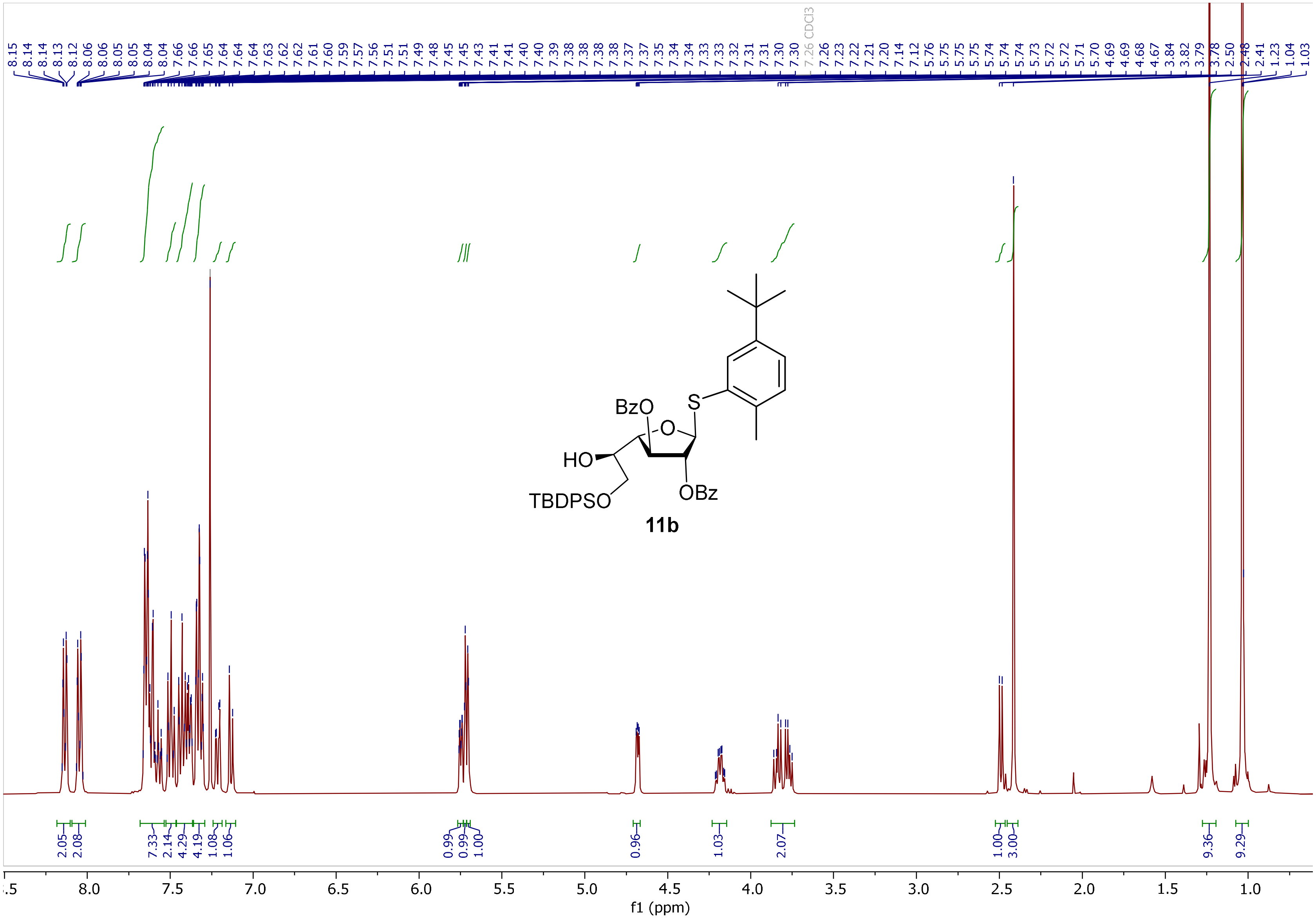

The ^{1}H Spectrum of Compound **11b** (400 MHz, $CDCl_3$)

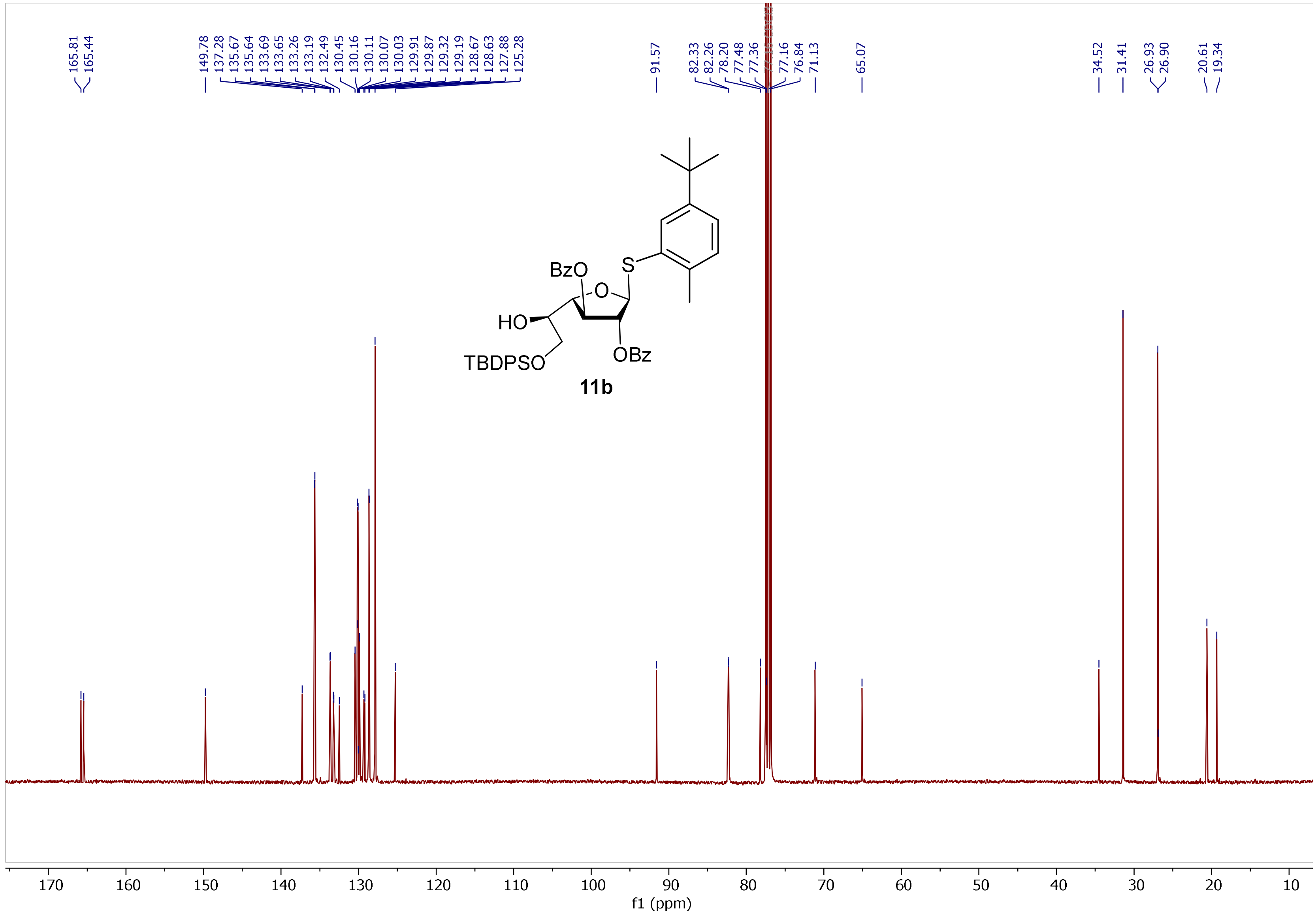

The $^{13}C\{^{1}H\}$ Spectrum of Compound **11b** (100 MHz, $CDCl_3$)

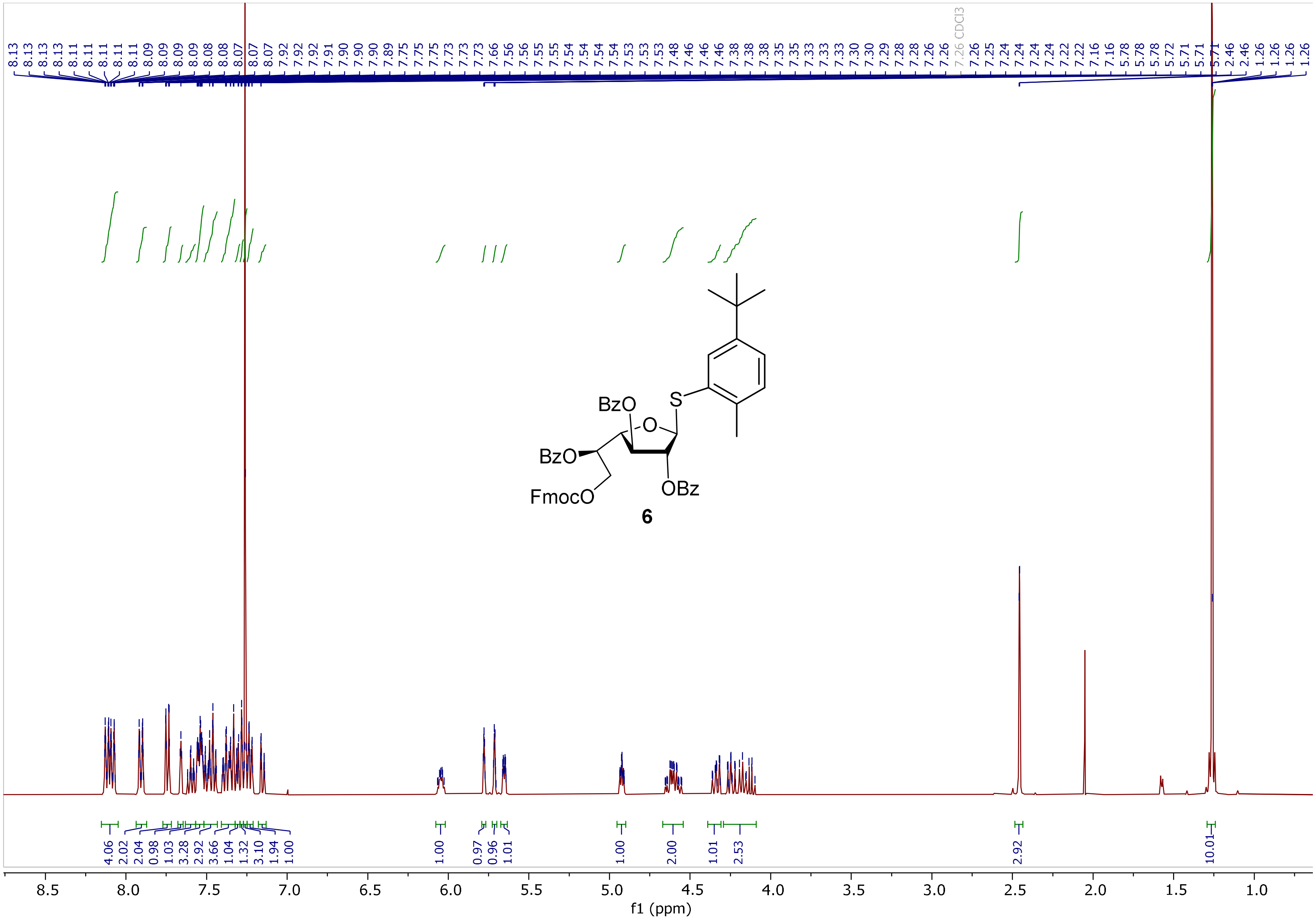

The [1]H Spectrum of Compound **6** (400 MHz, $CDCl_3$)

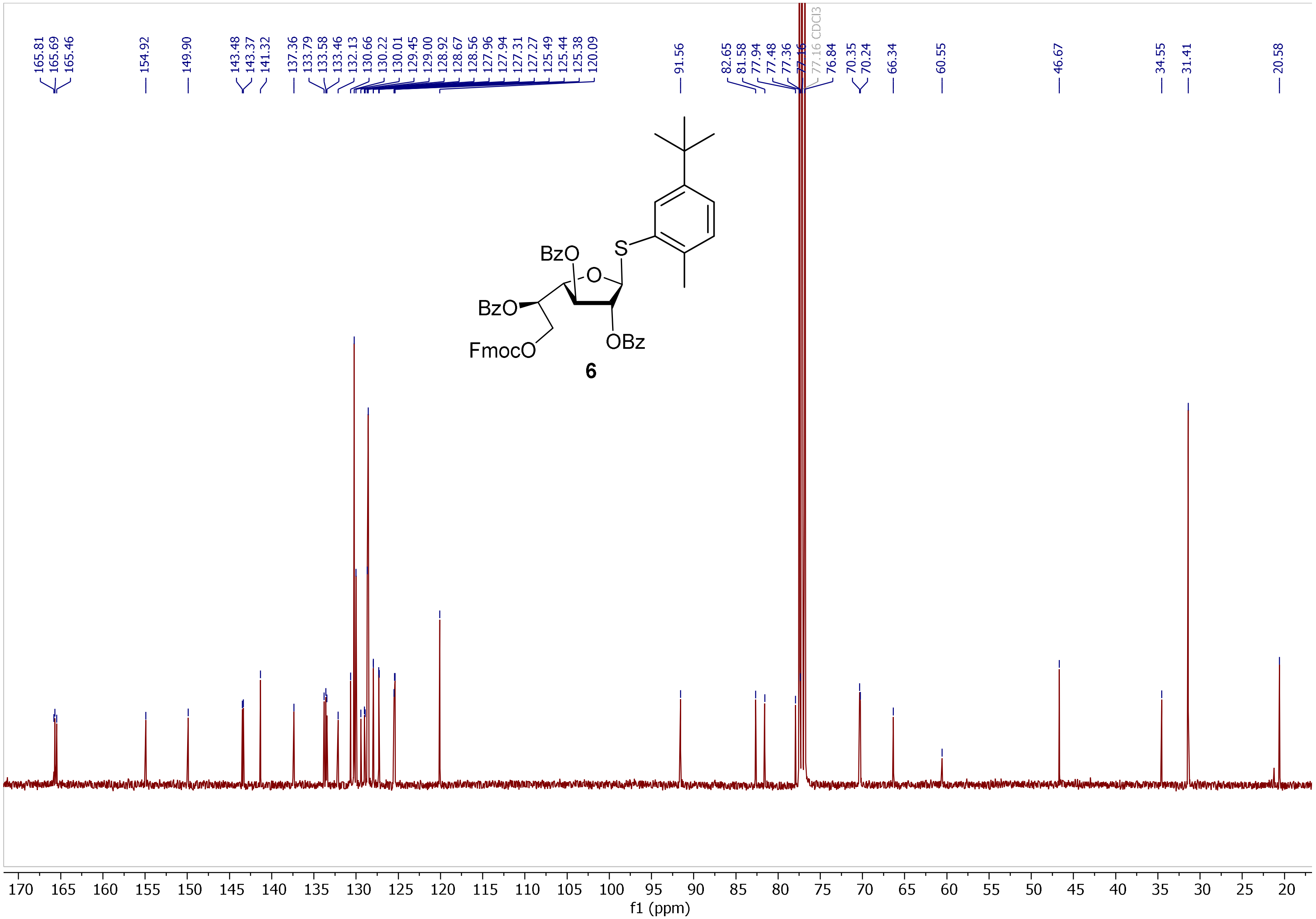

The $^{13}C\{^{1}H\}$ Spectrum of Compound **6** (100 MHz, $CDCl_3$)

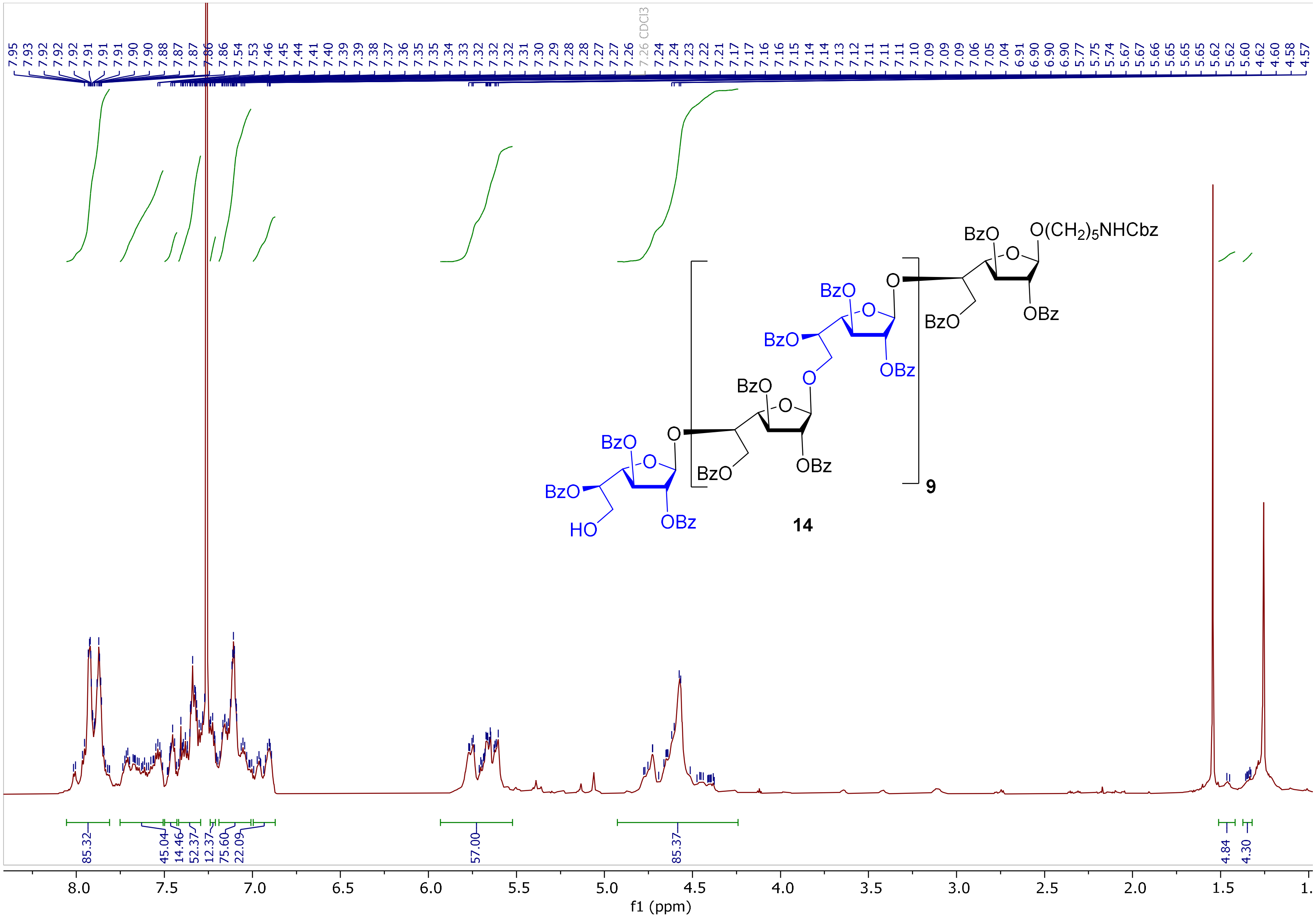

The ^{1}H Spectrum of Compound **14** (700 MHz, $CDCl_3$)

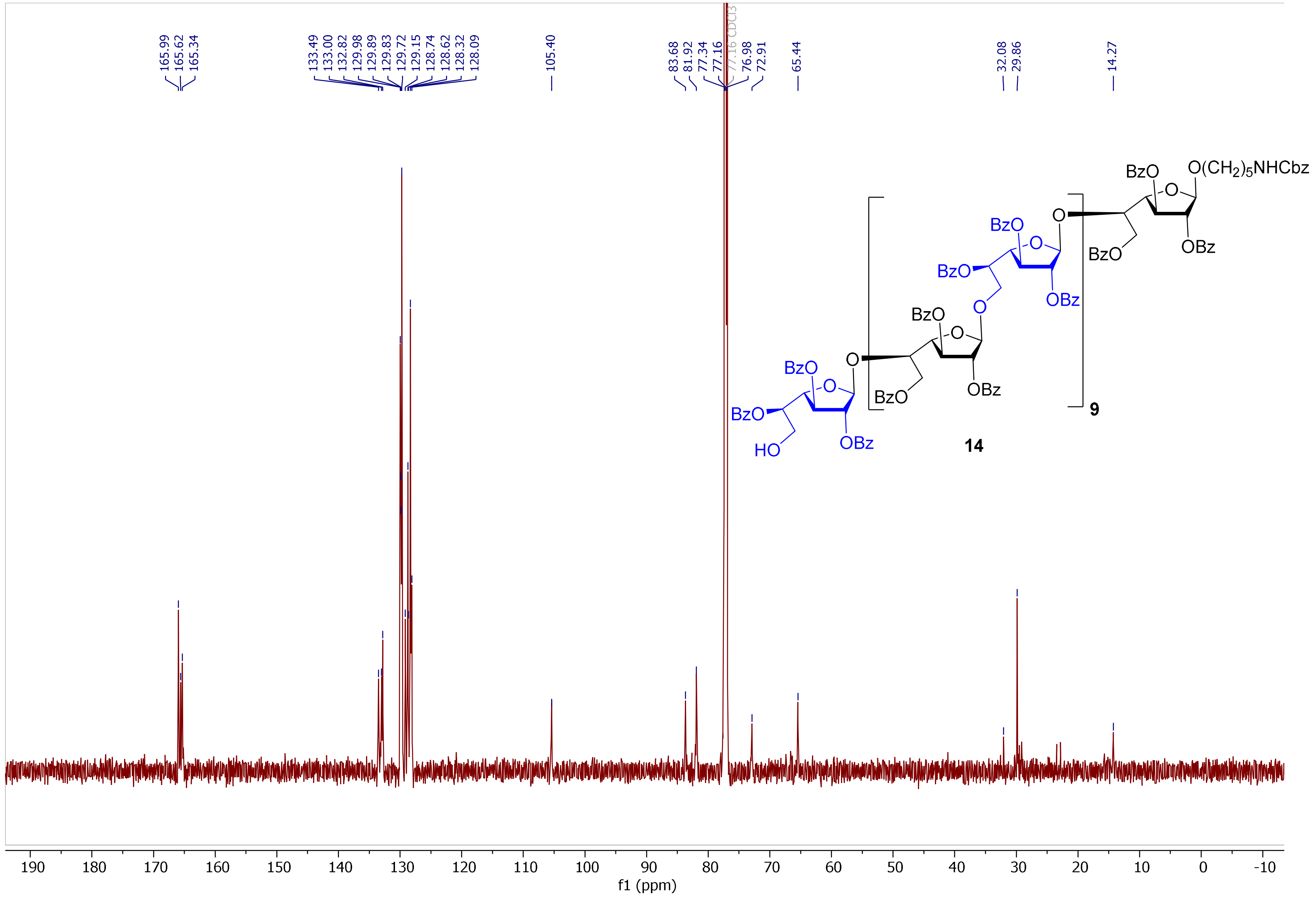

The $^{13}C\{^{1}H\}$ Spectrum of Compound **14** (175 MHz, $CDCl_3$)

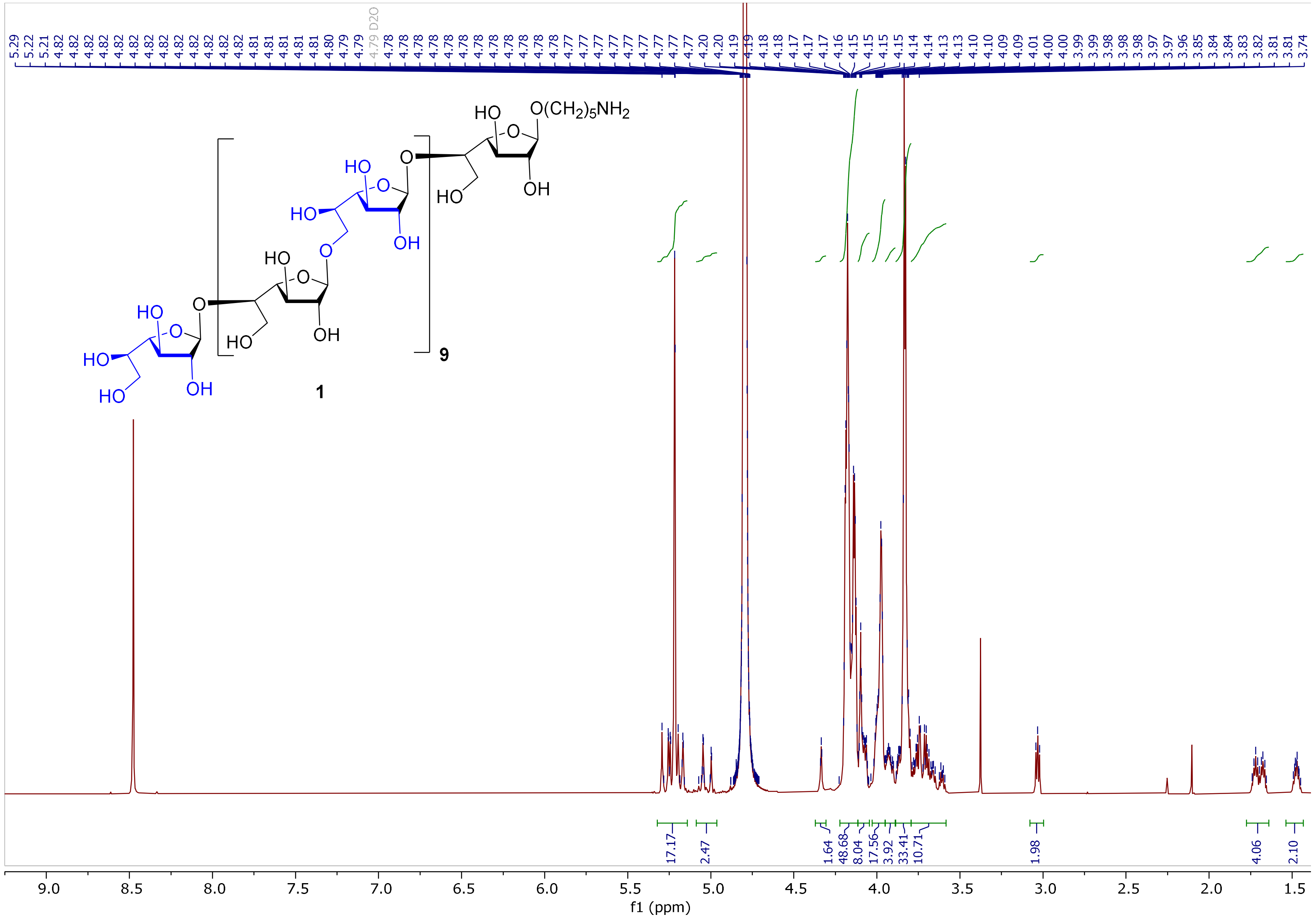

The ^{1}H Spectrum of Compound **1** (700 MHz, D_2O)

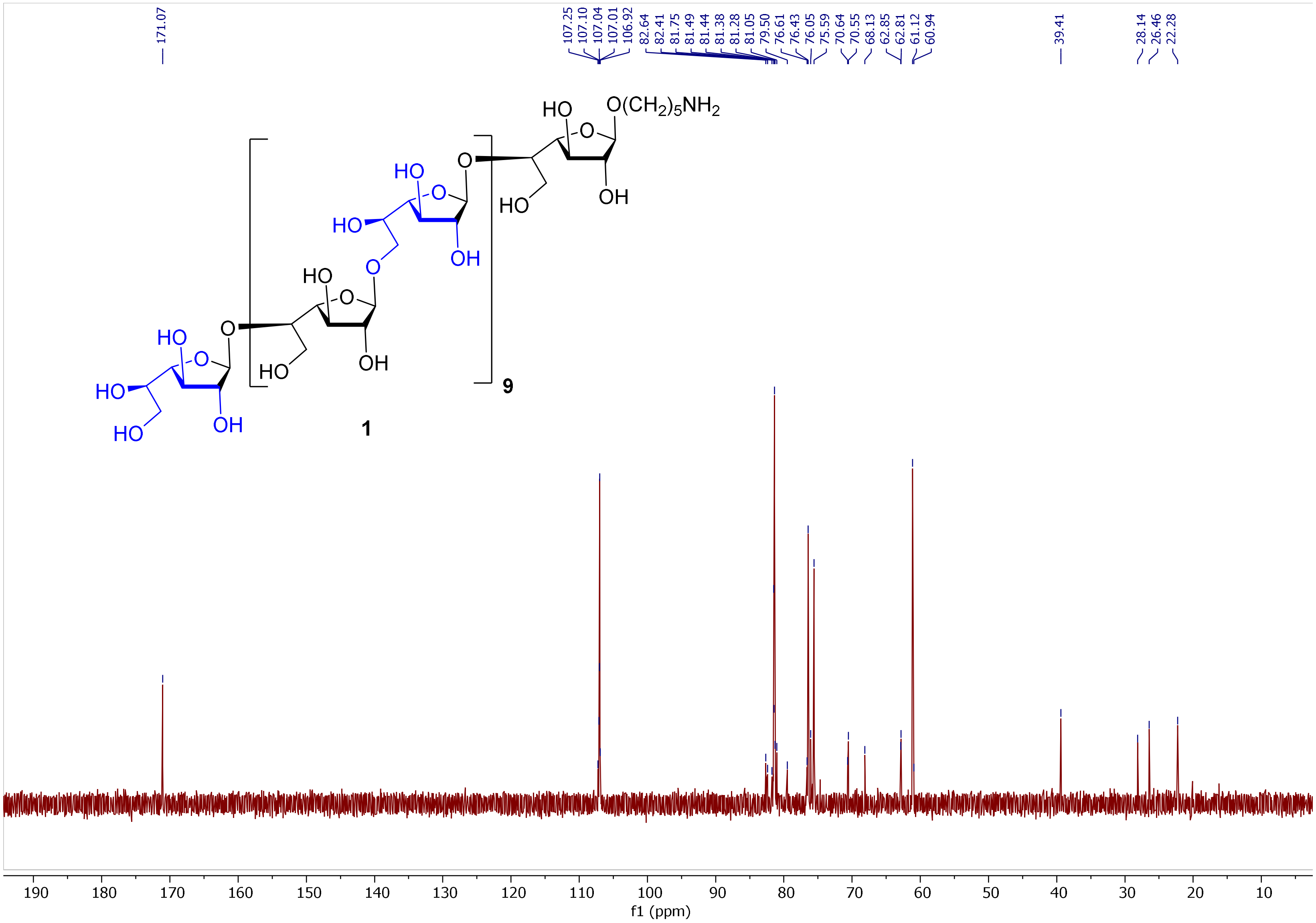

The $^{13}C\{^{1}H\}$ Spectrum of Compound **1** (175 MHz, D_2O)

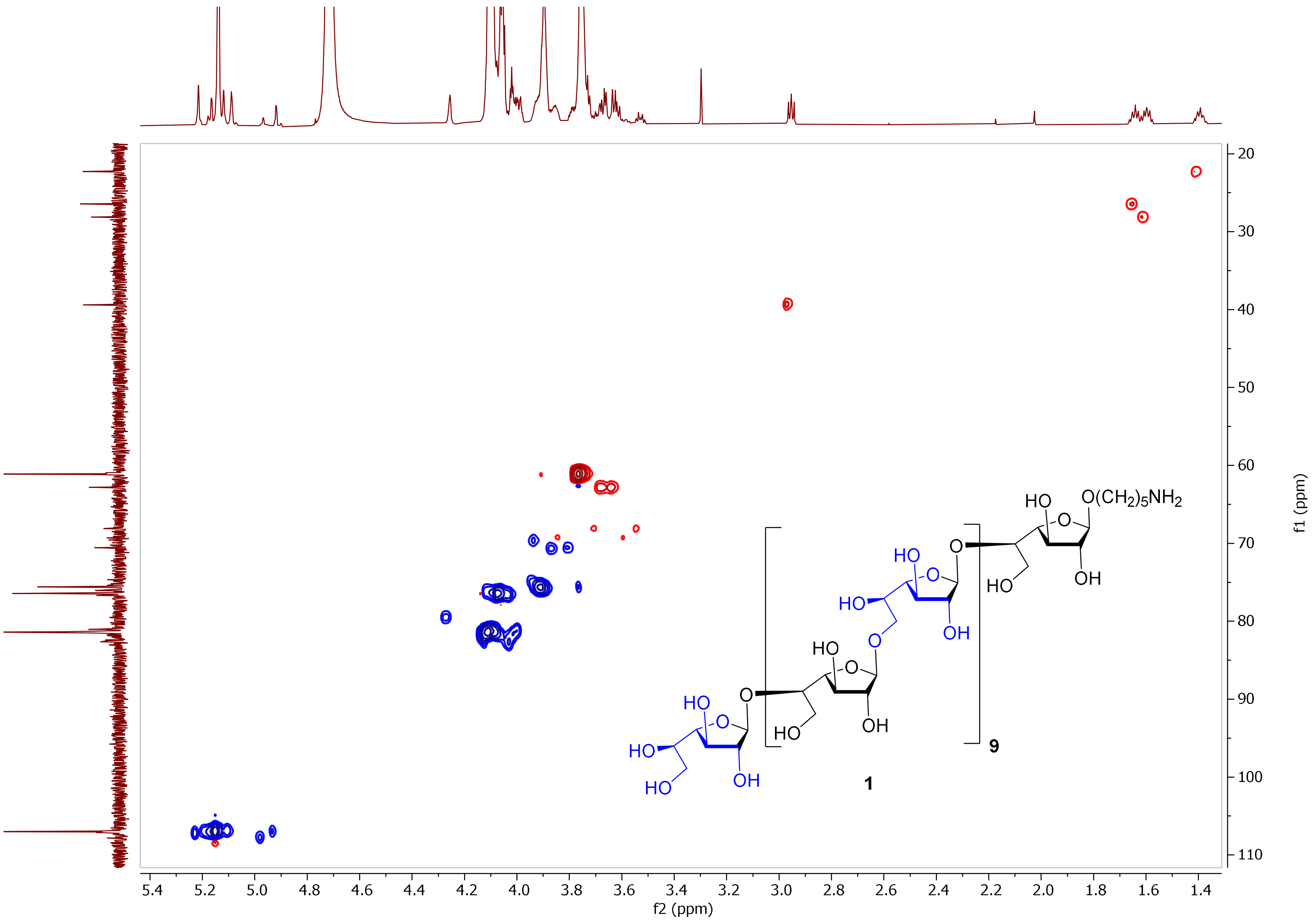

The HSQC Spectrum of Compound **1** (D_2O)
